# SPOP-mediated RIPK3 destabilization desensitizes LPS/sMAC/zVAD-induced necroptotic cell death

**DOI:** 10.1007/s00018-024-05487-7

**Published:** 2024-11-14

**Authors:** Ga-Eun Lee, Geul Bang, Jiin Byun, Weidong Chen, Dohyun Jeung, Hana Cho, Joo Young Lee, Han Chang Kang, Hye Suk Lee, Jin Young Kim, Kwang Dong Kim, Juan Wu, Soo-Bin Nam, Young Jik Kwon, Cheol-Jung Lee, Yong-Yeon Cho

**Affiliations:** 1 BK21-4th, College of Pharmacy, The Catholic University of Korea, 43, Jibong-ro, Wonmi-gu, Bucheon- si, Gyeonggi-do 14662 Republic of Korea; 2 Biopharmaceutical Research Center, Ochang Institute of Biological and Environmental Sciences, Korea Basic Science Institute, 162, Yeongudanji-ro, Ochang-eup, Cheongwon-gu, Cheongju-si, 28119 Republic of Korea; 3 Research Center for Bioconvergence Analysis, Korea Basic Science Institute, Ochang, Cheongju-si, Chungbuk, 28119 Republic of Korea; 4 College of Pharmacy, The Catholic University of Korea, 43, Jibong-ro, Wonmi-gu, Bucheon-si, Gyeonggi-do 14662 Republic of Korea; 5 Research Institute for Controls and Materials of Regulated Cell Death, The Catholic University of Korea, 43, Jibong-ro, Wonmi-gu, Bucheon-si, Gyeonggi-do 14662 Republic of Korea; 6 BK21-Four, Division of Applied Life Science, Gyeongsang National University, 501, Jinju-daero, Jinju- si, Gyeongsangnam-do 52828 Republic of Korea; 7 Department of Pharmaceutical Sciences, University of California, 132, Sprague Hall, Irvine, CA 92697 USA

**Keywords:** RIPK3, SPOP, Ubiquitination, Kinases, Necroptosis

## Abstract

RIPK1/RIPK3-MLKL signaling molecules are fundamental in initiating necroptotic cell death, but their roles in the development of colon cancer are unclear. This study reports that RIPK3 interacted with SPOP, a component of the E3 ligase within the Cul3 complex. This interaction leads to K48-linked ubiquitination and subsequent proteasomal degradation of RIPK3. Two distinct degron motifs, PETST and SPTST, were identified within the linker domain of RIPK3 for SPOP. RIPK3 phosphorylations at Thr403 by PIM2 and at Thr412/Ser413 by ERK2 are essential to facilitate its interaction with SPOP. Computational docking studies and immunoprecipitation analyses showed that these PIM2 and ERK2 phosphorylations bolster the stability of the RIPK3-SPOP interaction. In particular, mutations of RIPK3 at the degron motifs extended the half-life of RIPK3 by preventing its phosphorylation and subsequent ubiquitination. The deletion of SPOP, which led to increased stability of the RIPK3 protein, intensified LPS/sMAC/zVAD-induced necroptotic cell death in colon cancer cells. These findings underscore the critical role of the SPOP-mediated RIPK3 stability regulation pathway in controlling necroptotic cell death.

**Supplementary Information:**

The online version contains supplementary material available at 10.1007/s00018-024-05487-7.

## Introduction

Necroptosis, a form of regulated cell death, is characterized by the loss of cell plasma membrane integrity and the swelling of organelles, particularly mitochondria [1]. It is initiated by various stimuli, such as tumor necrosis factor, which activates receptor-interacting serine/threonine kinase 1 (RIPK1) and RIPK3 [2]. Typically, RIPK1 ubiquitination promotes NF-κB and MAPK activation, leading to cell survival, whereas deubiquitination triggers caspase-8-mediated apoptosis [3, 4]. When caspase-8 is inhibited (e.g., by carbobenzoxy-valyl-alanyl-aspartyl-[O-methyl]-fluoromethylketone, zVAD) or genetically depleted, RIPK1 interacts with RIPK3 via the C-terminal RIP homotypic interaction motif (RHIM) [5], leading to necroptosis [5]. Although excessive necroptosis contributes to various diseases, including, but not limited to, systemic inflammation, ischemic reperfusion injury, and neurodegeneration [6], it can sensitize cancer cells to anti-cancer drugs. Thus, modulating RIPK1/RIPK3 signaling may be advantageous for overcoming cancer.

Colorectal cancer (CRC) is a multifactorial disease with etiological relevance to genetic predispositions, environmental exposures, and inflammatory conditions of the intestinal tract [7]. Comparative analyses of human CRC tissues and adjacent normal tissues showed significantly elevated expression of RIPK1 levels in the cancerous tissues, correlating positively with the cancer progression stages and three-year mortality rates [8]. This suggests a potential oncogenic role for RIPK1. By contrast, other studies have indicated a marked decrease in RIPK1 and RIPK3 expression in human colon cancer tissues compared to the adjacent normal tissues [9]. This reduction has been attributed to the hypoxia-induced suppression of RIPK1 and RIPK3 rather than epigenetic DNA modifications [9]. Additional research corroborated the significant decrease in RIPK3 expression in human CRC tissues relative to the adjacent normal tissues [10–13], with this downregulation associated with adverse clinicopathological parameters, such as T stage, M stage, and AJCC stage [10], and impaired responses of colon cancer cells to necroptosis inducers [9]. In addition, mice deficient in RIPK3 exhibited heightened susceptibility to colitis-associated CRC and the increased production of tumor-promoting factors and pro-inflammatory mediators [11]. Patients exhibiting elevated levels of RIPK3 responded more favorably to 5-fluorouracil-based chemotherapy regimens, suggesting that RIPK3 expression may serve as a potential predictive biomarker for treatment response [14]. On the other hand, some studies reported conflicting observations, indicating increased levels of RIPK3 expression in human and mouse colonic cancers, including colitis-associated cancer (CAC) [4, 15–17]. These findings highlight the critical role of the RIPK3-MLKL signaling pathway in regulating necroptosis. Nevertheless, there is limited research on how the stabilities of signaling molecules within this pathway are regulated. Recent studies suggested that PELI1 and TRIM25 function as direct E3 ligases for RIPK3, promoting its ubiquitination and suppressing necroptosis [18, 19]. The molecular mechanisms controlling the subcellular localization of RIPK3, as revealed by immunocytofluorescence and western blotting [18, 20], and the inhibition of its ubiquitination through lysine (K) to alanine (A) or arginine (R) substitutions, suggest the involvement of an additional E3 ligase in RIPK3 ubiquitination. Hence, other regulatory factors, potentially an unknown E3 ligase, may play a role in the ubiquitination and functional regulation of RIPK3. Moreover, RIPK3 is a nucleocytoplasmic shuttling protein [21] that facilitates necroptosis occurrence [22]. These findings suggest that the molecular mechanisms regulating RIPK3 protein stability have been understudied. Overall, these results strongly suggest that E3 ligase(s) responsible for RIPK3 ubiquitination and destabilization may also exist in the nucleus, playing a role in its regulation within different cellular compartments.

Speckle-type POZ protein (SPOP) is an adaptor protein for E3 ubiquitin ligases that is frequently mutated in a wide range of cancers, including prostate, breast, endometrial, liver, and colon cancers [23]. SPOP downregulation has been linked to colorectal cancer (CRC), with its reduced mRNA or protein levels significantly associated with poor differentiation, distant metastasis, and advanced TNM stages [24]. Nevertheless, the role of SPOP in CRC has not been studied extensively. Currently, only one SPOP mutation has been found in a cohort of 45 colorectal cancer patients by the mutational landscape study of SPOP [23, 25]. Importantly, although SPOP mutations are barely detected in colorectal cancer, SPOP downregulation at the mRNA or protein levels is frequently observed in 20–61% of colorectal cancer patients [23–27]. These results suggest that SPOP-mediated target protein destabilization may be crucial in colorectal cancer development, but the precise molecular mechanisms underlying how SPOP regulates the stability of its target proteins are unclear.

This study reports a novel interaction between RIPK3 and SPOP. This interaction induced RIPK3 ubiquitination and accelerated RIPK3 degradation via the proteasomal degradation pathway. Importantly, SPOP and RIPK3 interactions are required for RIPK3 phosphorylation at the two degron motifs located in the linker domain by PIM2 and ERK2. In particular, the increased RIPK3 level by SPOP depletion sensitized LPS/sMAC/zVAD-induced necroptosis in HT-29 colon cancer cells. Overall, RIPK3 stability regulation by SPOP plays an essential role in necroptosis sensitization.

## Materials and methods

### Reagents

Dimethyl sulfoxide (Cat #: D2650), cycloheximide (Cat #: 01810), and MG132 (Cat #: C2211) were purchased from Sigma–Aldrich (Sigma–Aldrich Korea, Gangnam, Seoul, Korea). Antibodies including anti-RIPK3 (Cat #: 13526 and 10188), anti-phospho-RIPK3 (Ser277) (Cat #: 93654), and anti-Cullin3 (Cat #: 2759) were acquired from Cell Signaling Technology (Danvers, MA, USA). Anti-SPOP (Cat #: 16750-1-AP) was obtained from Proteintech (Rosemont, IL, USA). Anti-phospho-MLKL (S358) (Cat #: ab187091) was supplied by Abcam (Abcam Korea, Hanam-city, Gyeonggi-do, Korea). Anti-Myc (Cat #: sc-40), anti-Myc-HRP (Cat #: sc-40-HRP), anti-GST (Cat #: sc-138), and anti-β-actin (Cat #: sc-47778) antibodies were purchased from Santa Cruz Biotechnology (Dallas, TX, USA). Anti-DDDDK-tag (as known as Flag) (Cat #: M185), anti-DDDDK-tag-HRP (known as Flag-HRP) (Cat #: M185-7), and anti-HA-HRP (Cat #: M180-7) antibodies were procured from MBL International Corporation (Woburn, MA, USA). Protein G Sepharose beads (Cat #: 17-0618-02) were purchased from GE Healthcare (Chicago, IL, USA). Glutathione Separopore^®^ 4B (Cat #: 20181050) was supplied by BioWORLD (Dublin, OH, USA). HisPur™ Ni-NTA Resin (Cat #: 88221) and CyQUANT™ LDH Cytotoxicity Assay Kit (Cat #: C20300) were obtained from Thermofisher Scientific (Waltham, MA, USA). Lambda Protein Phosphatase (Lambda PP) was obtained from New England Biolabs (NEB) (Ipswich, England, UK). Cell Counting Kit-8 (CCK-8) (Cat #: CK-04) was purchased from DOJINDO Molecular Technologies (Rockville, Maryland, USA). The lipopolysaccharides from Escherichia coli O111:B4 (Cat #: L2630) were procured from Sigma–Aldrich (Sigma–Aldrich Korea, Gangnam, Seoul). zVAD-FMK (Cat #: S7023) and LCL161(as known as sMAC mimetic) (Cat #: S7009) were purchased from Selleckchem (Houston, TX, USA).

### Cell culture

HEK293T cells and HeLa cells were cultured in Dulbecco’s Modified Eagle’s Medium (DMEM, Cat #: 10-013-CV, Corning Korea, Seoul) supplemented with 10% fetal bovine serum (FBS, Cat #: 35-015-CV, Corning Korea, Seoul). The HT-29 colorectal cancer cells were cultured in RPMI-1640 (Cat #: 10-040-CV, Corning Korea, Seoul) supplemented with 10% FBS. All cells were incubated at 37 ℃ in a 5% CO_2_ incubator. The cells were fed fresh medium every other day and passaged upon reaching approximately 90% confluence.

### Expression vectors

The pCMV-Myc- and HA-fusion expression vectors were obtained from TAKARA Bio Inc. (Kusatsu, Shiga, Japan). The pcDNA4-His/Max fusion vector was supplied by Thermo Fisher Scientific. The pCMV-Flag- and pEBG-GST-tag expression vectors were purchased from Addgene (Watertown, TX, USA), while the pGEX-5X-1 bacterial expression vector came from Amersham (GE Healthcare, Chicago, IL, USA). The expression vectors for SPOP, RIPK3, PIM1/2, ERK1, and RIPK3 mutants were constructed by cloning the DNA fragments into the pCMV-Myc, -Flag, -HA, or pEBG-GST vectors. Stable cell lines were established by a lentiviral infection using the pCDH-CMV-MCS-EF1-Puro vectors, and SPOP depletion was performed using Crispr-Cas9 (pLenti-CRISPRv2). All vectors were validated by DNA sequencing before use.

### Gene knockdown and ectopic expression

Viral particles were produced by the co-transfection of a lentiviral expression vector and packaging vectors. Briefly, HEK293T cells (1 × 10^6^ cells/100-mm dish) were seeded and co-transfected with the lentiviral expression vector and packaging vectors. The viral particles were harvested at 48 h by filtering the culture supernatant through a 0.45 μm acetate syringe filter and used to infect different cells by 24 h culturing in complete medium containing 1 µg/ml polybrene (Sigma–Aldrich). The infected cells were discriminated from non-infected cells by 24 h culturing in a complete medium containing puromycin (3 µg/ml). The efficiency of knockdown or overexpression was confirmed by western blotting using specific antibodies as indicated.

### Cell proliferation assay

Cell proliferation was assessed by seeding HT-29 cells (2 × 10^3^) infected with either mock or Crispr-Cas9-sgSPOP into 96-well plates and cultured overnight. The next day (0 h), cell proliferation was measured by adding 10 µl of CCK-8 into each well, followed by 1 h incubation at 37 ℃ in a CO_2_ incubator, according to the manufacturer’s protocols (DOJINDO molecular technologies, Rockville, MD, USA). The absorbance was then measured at 450 nm using an xMark microplate spectrophotometer (Bio-Rad Laboratories, Hercules, California, USA).

### Immunoprecipitation

Protein–protein interactions were evaluated by immunoprecipitation (IP) using cell lysates (300–400 µg) stably or transiently expressing tag-fusion proteins. Briefly, the lysates were incubated overnight at 4 °C with 30 µl of 50% protein G-Sepharose slurry and 2 µg of the appropriate antibodies. After washing with a washing buffer (20 mM Tris at pH 8.0, 100 mM NaCl, 1 mM EDTA, and 0.5% NP-40) to remove the non-specific binding, the bound proteins were detected by western blotting. For endogenous proteins, 1 mg of cell lysates was hybridized with 5 µg of pre-immune or SPOP antibodies, followed by overnight binding and subsequent detection, as described above.

### Immunocytofluorescence assay

HT-29 cells (2 × 10^4^) stably expressing mock or sgSPOP were seeded into four-chamber slides (Falcon, Corning, NY, USA), incubated overnight, and treated with LPS/sMAC/zVAD cocktail (refer to as LSZ: 10 ng/ml LPS, 100 nM of sMAC, and 20 µM zVAD) for 6 h. The cells were fixed with 4% formaldehyde, permeabilized with 0.5% of Triton X-100, blocked with 1× PBS/0.02% Tween 20/1% BSA at 37 °C for 1 h, and then incubated with the appropriate antibodies by gentle rocking at 4 ℃. Endogenous phospho-RIPK3 at Ser227 and -MLKL at Ser358 proteins were visualized using secondary antibodies that are conjugated Alexa-488 or -568 under a confocal microscope (LSM 710 laser scanning confocal microscopy, Carl Zeiss, Oberkochen, Germany).

### Cell death sensitivity assay

The effects of SPOP depletion on the cell death sensitivity via necroptosis were measured by seeding HT-29 cells (7.5 × 10^3^) stably expressing mock or sgSPOP into 96-well plates, culturing overnight, and treating with LPS/sMAC/zVAD cocktail for the indicated time. The LDH activity was measured by harvesting the culture media and measuring the LDH activity by spectrophotometry (absorbance at 490 nm and 680 nm) using a CyQUANT™ LDH Cytotoxicity Assay kit (Invitrogen, Waltham, MA, USA) according to the manufacturer’s protocols.

### Flow cytometry analysis

Flow cytometry analysis was conducted to evaluate the cell death pathways. Briefly, HT-29 cells (5 × 10^5^) stably expressing mock or sgSPOP were seeded into 60-mm culture dishes, incubated overnight, and treated with LSZ (10 ng/ml lipopolysaccharide, LPS; 100 nM sMAC; and 20 µM zVAD) for indicated times. At each time point, the cells were harvested using 0.1% trypsin/EDTA buffer, washed, and stained with Annexin V and propidium iodide (1 µg/ml). The cell population in each sample was measured by flow cytometry using a FACSCalibur™ flow cytometer (BD, Franklin Lakes, NJ, USA).

### Quantification of mRNA

The total RNAs extracted from HT-29 cells stably expressing sh-mock or -SPOP were used to synthesize the cDNA by Bio-Rad iScript™ cDNA Synthesis Kit (Cat #: 1708890). The cDNAs were used to amplify RIPK3 and β-actin, an internal control, and evaluate the off-target effect of sh-SPOP on the RIPK3 mRNA levels using a polymerase chain reaction (PCR). The amplified DNAs were visualized by agarose gel electrophoresis and ethidium bromide staining using a ChemiDoc XRS^+^. The PCR primers for RIPK3 and β-actin amplification are as follows: RIPK3, sense 5’- CAAGGAGGGACAGAAATGGA-3’, antisense 5’- GCCTTCTTGCGAACCTACTG-3’; β-actin: sense 5’-CAGGTCATCACCATTGGCAATGAGC-3’, antisense 5’-GATGTCCACGTCACACTTCATGA-3’.

### In vitro ubiquitination assays

An in vitro ubiquitination assay was conducted to validate the SPOP-mediated RIPK3 ubiquitination. HEK293T cells (3 × 10^6^) were seeded into 100-mm culture dishes, cultured overnight, and transfected with Myc-SPOP. The Myc-SPOP proteins were collected for the in vitro ubiquitination assay by Myc immunoprecipitation. The bacterial purified GST-RIPK3 protein (200 ng) was used as a SPOP substrate for the in vitro ubiquitination assay. E1, E2 (UbcH3 and Ubc5a), and Biotin-Ubi were incubated together according to the manufacturer’s protocol (Ubiquitinylation kit, Enzo Life Science, NY, USA). The reaction was performed at 37 ℃ for 1 h and quenched by adding 6× loading buffer and boiling for 5 min. The ubiquitinated RIPK3 was detected by western blotting.

### Phos-tag gel electrophoresis

Phos-tag gel electrophoresis was conducted to evaluate the RIPK3 phosphorylation. Briefly, HEK293T cells (3 × 10^6^) were seeded into 100-mm culture dishes, cultured overnight, and transfected with plasmid as indicated. After 24 h cultivation, the cells were treated for 5 h with the indicated chemical inhibitors, harvested, and lysed with NP-40 lysis buffer. The whole cell lysates (30 µg) were loaded onto the phos-tag gel (SuperSep™ Phos-tag™, Cat #: 198-17981, Fuji film, Osaka, Japan) and electrophoresed. The gels were washed three times in 10 mM EDTA/Transfer buffer with agitation for 20 min, re-soaked into EDTA-free transfer buffer for 10 min, and blotted onto the PVDF membrane. The bands were visualized using the specific antibodies, as indicated.

### Protein purification

BL21 bacteria transformed with the pGEX-5X-1-RIPK3-wildtype (RIPK3-wt) expression vector were induced to express RIPK3-wt using 0.1 mM IPTG (isopropyl β-D-1-thiogalactopyranoside). The expressed RIPK3-wt protein was partially purified from glutathione-conjugated beads by eluting with 10 mM reduced glutathione. The purified proteins were verified by western blotting, using specific antibodies against glutathione S-transferase and RIPK3.

### Computational docking between SPOP and RIPK3

Computational protein docking was performed using Discovery Studio (Ver. 2021) computer software and processed as described elsewhere [28]. The crystal structure of SPOP (PDB ID: 3HQI) was retrieved from the Protein Data Bank (https://www.rcsb.org/). The structure of RIPK3, covering amino acids 1–425, was modeled using the “Build Homology Models” tool in Discovery Studio, based on the mouse RIPK3 structure (PDB ID: 4M66), which shared 64% sequence similarity with human RIPK3. The complex structural model of SPOP and RIPK3 was performed using an in silico receptor-ligand docking system. A rigid-body docking program, ZDOCK [29], with the default parameters, was used to search all possible binding modes for the antibody and antigen to obtain more accurate predictions for the two proteins by evaluating the shape complementarity and desolvation energy. The leading 61 predictions from ZDOCK were fed into RDOCK, where they were minimized by CHARMm to improve the energy levels and eliminate clashes. The desolvation energy and electrostatics were recomputed using RDOCK. A reliable complex structure in the solvated system was obtained by simulating the best prediction from RDOCK with water molecules and optional counterions to model the solvent and minimize and calculate the solvation model in the Discovery Studio program. The solvated system can be optionally minimized to eliminate van Der Waals clashes. Finally, the interaction between the MATH domain of SPOP and the degron motifs of the linker domain in RIPK3 was emulated using the complementarity-determining regions (CDRs) of the rational antibody-antigen complex model. The rational antibody-antigen complex model satisfied the complementarity-determining regions (CDRs) of the MATH domain of SPOP and the degron motifs of the linker domain in RIPK3.

## Results

### Cullin 3-composed E3 ligase regulates RIPK3 stability

**Fig. 1:**
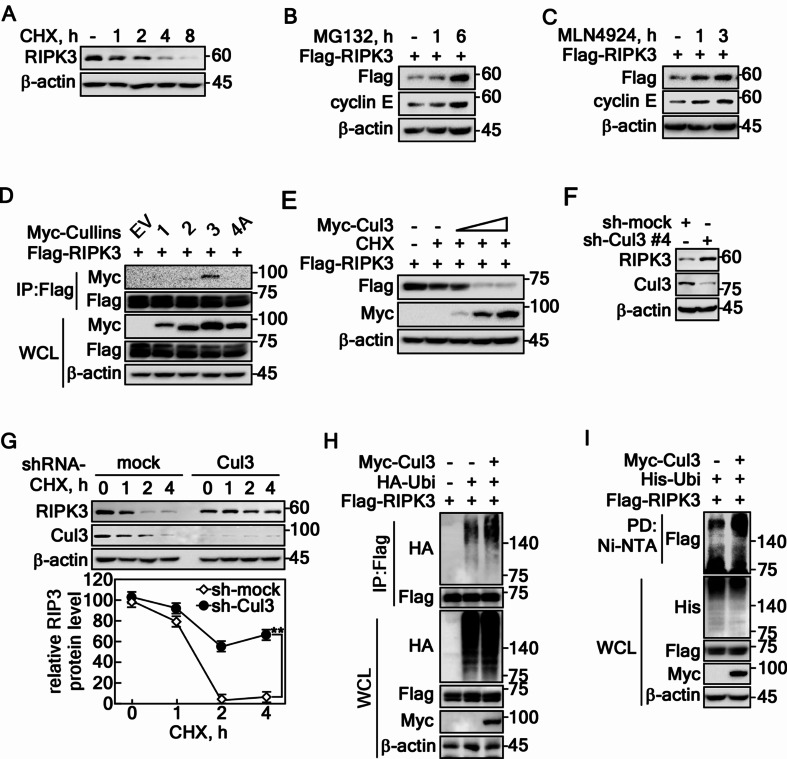
Cullin3-composed E3 ligase regulates RIPK3 stability. **A** RIPK3 protein degradation was evaluated by cycloheximide (10 µg/ml) in HT-29 cells in a time-dependent manner. **B** The recovery of RIPK3 was confirmed in HeLa cells treated with MG132 (10 µM) in a time-dependent manner. **C** Cullin inhibition results in an increased level of RIPK3 protein in HeLa cells. **D** The specific interaction between RIPK3 and Cul3 was confirmed in HEK293T cells. **E** Cul3 overexpression suppresses the RIPK3 protein levels in a dose-dependent manner in HEK293T cells. **F** Increased RIPK3 protein level was confirmed by the knockdown of Cul3 using sh-Cul3 in HT-29 cells. **G** RIPK3 half-life was prolonged by sh-Cullin 3 in HT-29 cells. The error bars obtained from three independent experiments indicate the SEM. ** *p* < 0.01 (Student *t*-test). **H** IP confirmed the Cul3-mediated increased RIPK3 ubiquitination of RIPK3 in HEK293T cells. **I** Cul3-mediated increased RIPK3 ubiquitination was confirmed by the pulldown of ubiquitin in HEK293T cells. **A-I** β-actin was used as an internal control for an equal protein loading

Previous studies indicated that the role of RIPK3 in cancer cell survival and death is context-dependent. Research on necroptosis has focused primarily on regulating the RIPK3-MLKL signaling pathway, leaving the mechanisms of RIPK3-mediated necroptosis, particularly through protein stability regulation, but it is less understood. Leveraging the expertise in protein stability via F-box proteins [13, 28, 30], cycloheximide (CHX), a translation inhibitor, reduced the RIPK3 levels (Fig. 1A) while MG132, a proteasomal degradation inhibitor, increased RIPK3 levels (Fig. 1B). This suggests that regulating the RIPK3 stability could be a key mechanism controlling its activity. The involvement of the cullin family in regulating RIPK3 protein stability was confirmed using MLN4924, a cullin neddylation inhibitor, which increased the RIPK3 levels (Fig. 1C). Immunoprecipitation (IP) with cullin 1, 2, 3, or 4 A showed that RIPK3 specifically interacts with cullin 3 (Cul3) (Fig. 1D). Moreover, decreasing the RIPK3 protein levels by increasing the Cul3 doses showed that a Cul3-composed E3 ligase plays a key role in RIPK3 stability regulation (Fig. 1E). In contrast, Cul3 knockdown increased the RIPK3 protein level (Fig. 1F). Moreover, Cul3 knockdown cells exhibited a prolonged RIPK3 half-life compared to mock control cells (Fig. 1G), suggesting that Cul3 complex induces RIPK3 destabilization. Specifically, increased RIPK3 ubiquitination by Cul3 co-overexpression was observed by IP/western blotting using RIPK3 and His-Ubi antibodies (Fig. 1H) and by pulldown/western blotting experiments using Ni-NTA and His-Ubi antibody (Fig. 1I). These results showed that the E3 ligase composing Cul3 complex destabilizes RIPK3 protein via ubiquitination.

### SPOP-induced RIPK3 K48 ubiquitination reduces RIPK3 half-life

**Fig. 2:**
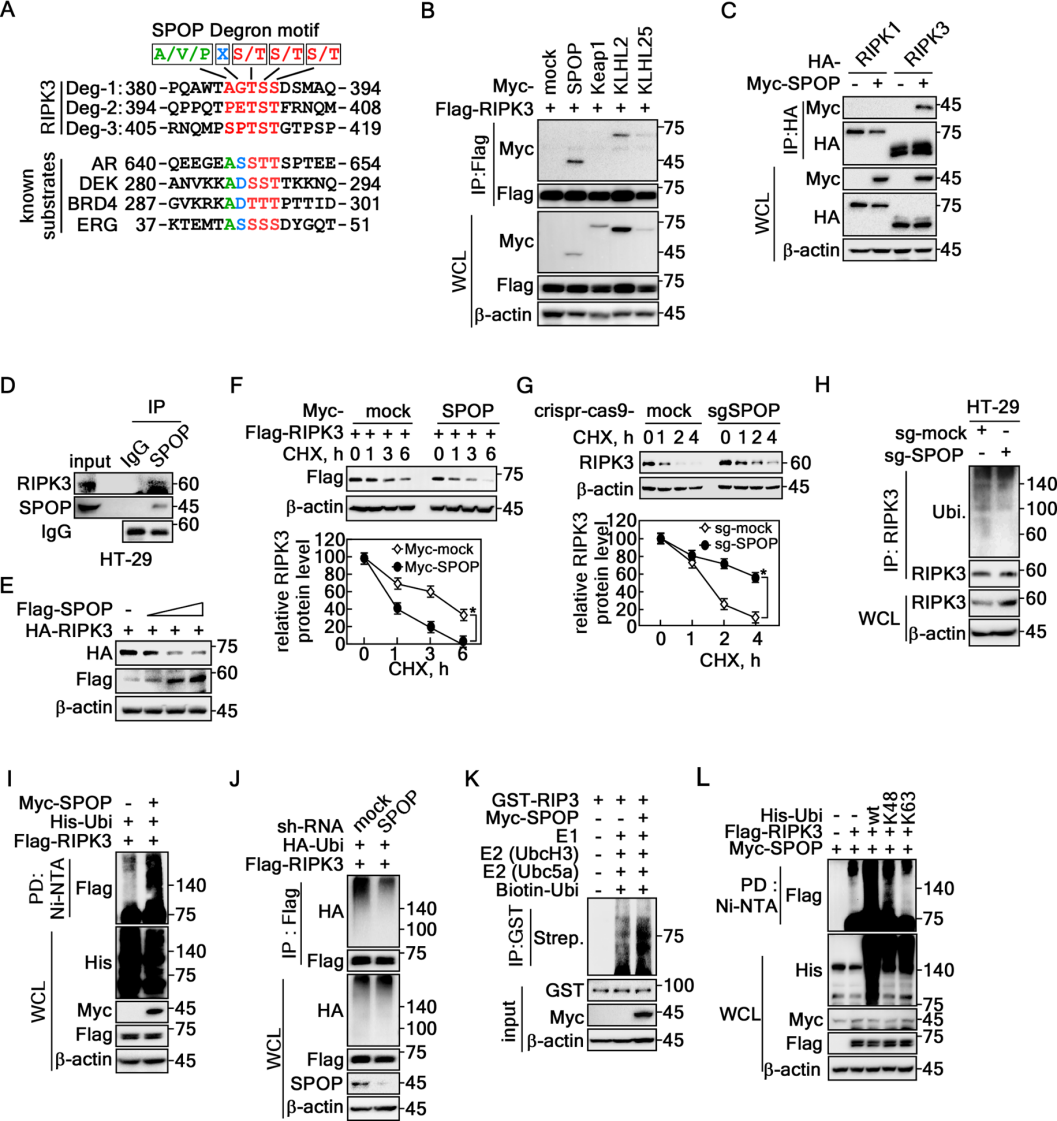
SPOP-induced RIPK3 K48 ubiquitination reduces RIPK3 half-life. **A** Amino acid sequence analysis of the RIPK3 showed putative degron motifs for the SPOP, A/V/P-X-S/T-S/T-S/T. **B** Screening of RIPK3 interaction with cullin 3 composing E3 ligases by IP in HEK293T cells. **C** Specific interaction between RIPK3 and SPOP was confirmed by IP in HEK293T cells. **D** IP confirmed the endogenous interaction of RIPK3 with SPOP in HT-29 cells. **E** RIPK3 protein levels were decreased by SPOP expression in a dose-dependent manner in HEK293T cells. **F ***Top panels*, RIPK3 half-life was shortened by the coexpression of SPOP in HeLa cells. *Graphs*, Normalized RIPK3 band intensities corresponding β-actin intensity. **G ***Top panels*, Knockout SPOP by Crispr-Cas9-SPOP extended RIPK3 half-life in HT-29 cells. *Graphs*, Normalized RIPK3 band intensities corresponding β-actin intensity. **H** RIPK3 ubiquitination in the endogenous levels was decreased in Crispr-Cas9-SPOP knockout HT-29 cells. **I** SPOP overexpression induced RIPK3 ubiquitination in HEK293T cells. **J** SPOP knockdown abolished RIPK3 ubiquitination in HEK293T cells. **K **In vitro RIPK3 ubiquitination assay. **L** Confirmation of the K48-ubiquitination of RIPK3 was mediated by SPOP in HEK293T cells. **B-C** and **E-L** β-actin was used as an internal control for an equal protein loading. **F** and **G** The error bars from three independent experiments indicate the SEM. * *p* < 0.05 (Student *t*-test).

An amino acid sequence search for the consensus degron motifs against Cul3 E3 ligase members suggests that RIPK3 harbored potential degron motifs, Ф-П-S-S/T-S/T (Ф: nonpolar; П: polar) (Fig. 2A), for SPOP [31]. The IP with Cul3 complex members, including SPOP, Keap1, KLHL2, and KLHL25, showed that RIPK3 interacted strongly with SPOP and relatively weakly with KLHL2 (Fig. 2B). The interaction of SPOP with RIPK3, not RIPK1 (Fig. 2C), suggesting that SPOP may act as a specific E3 ligase for RIPK3. Based on the levels of RIPK3 and SPOP endogenous expression in colon cancer cell lines, including HT-29, HCT115, DLD-1, HCT116, WiDr, and SW480 (Supplementary Fig. 1A), HT-29 cells were selected for further experiments because they exhibited detectable levels of RIPK3 and SPOP, making them suitable for the present study. The endogenous IP using an SPOP antibody showed co-IP of RIPK3 (Fig. 2D), suggesting that SPOP and RIPK3 interactions occur under physiological conditions. The RIPK3 protein level was decreased gradually by SPOP coexpression in a dose-dependent manner (Fig. 2E). In particular, the RIPK3 half-life was shortened by SPOP coexpression (Fig. 2F, Supplementary Fig. 1B). In contrast, the depletion of SPOP by knockdown using sh-RNA-SPOP (sh-SPOP) (Supplementary Fig. 1C, D) or knockout by sg-SPOP (Fig. 2G, Supplementary Fig. 1E) prolonged the half-life of RIPK3 protein compared to the mock control groups. As expected, SPOP depletion suppressed the endogenous RIPK3 ubiquitination level (Fig. 2H). Ubiquitinated RIPK3 was increased by SPOP coexpression compared to the mock control vector (Fig. 2I), while RIPK3 ubiquitination was abolished by SPOP knockdown (Fig. 2J). Importantly, the in vitro ubiquitination assay confirmed the SPOP-mediated RIPK3 ubiquitination (Fig. 2K). In particular, SPOP-mediated RIPK3 ubiquitination was K48-linked ubiquitination, which is the leading protein degradation [32, 33], not K63-linked ubiquitination (Fig. 2L), which is involved in cell signaling [34, 35]. These results showed that SPOP-mediated RIPK3 K48-linked ubiquitination enhances RIPK3 destabilization.

### MATH domain of SPOP interacts with two degron motifs of RIPK3 in the linker domain

**Fig. 3:**
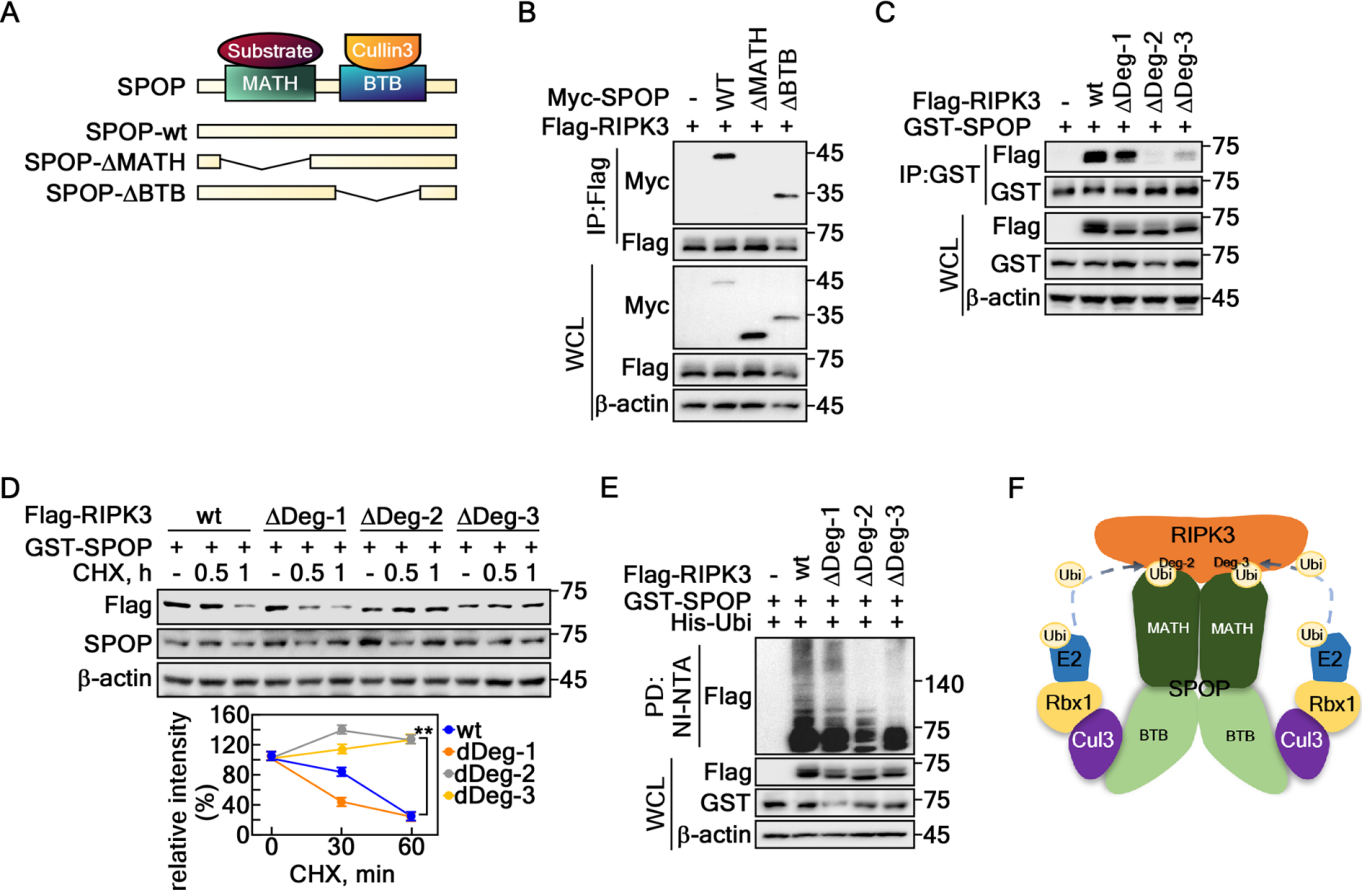
MATH domain of SPOP interacts with two degron motifs at the linker domain. **A** Schematic maps of the SPOP deletion mutants, SPOP-wt, SPOP-ΔMATH, and SPOP-ΔBTB. **B** MATH domain of SPOP interacted with RIPK3 in HEK293T cells. **C** SPOP recognized Deg-2 and -3 of RIPK3. ΔDeg-1, deleted aa 385–389; ΔDeg-2, deleted aa 399–403; and ΔDeg-3, deleted aa 409–413 by IP in HEK293T cells. **D** The prolonged half-life of RIPK3-ΔDeg-2 and -3. *Upper panels*, Confirmation of the prolonged half-life of RIPK3-ΔDeg-2 and -3 in HeLa cells. *Graph*s, The band intensities of RIPK3 by normalization using the β-actin band intensity. Densitometric computer program: NIH Image J (Ver. 1.42). The error bars from three independent experiments indicate the SEM. ** *p* < 0.01 (Student *t*-test). **E** SPOP-mediated RIPK3 ubiquitination was decreased in RIPK3-ΔDeg-2 and -3 detected by a Ni-NTA pulldown assay in HEK293T cells. **F** A proposed binding model of SPOP E3 ligase complex and RIPK3. **A** and **C-E.** β-actin was used as an internal control for an equal protein loading

MATH or BTB domain deletion mutants were constructed to identify the interaction domain mediating interactions between SPOP and RIPK3 (Fig. 3A). The IP using these mutated proteins showed that RIPK3 and SPOP interaction was mediated via the MATH domain of SPOP, not BTB (Fig. 3B). Based on the amino acid sequence analysis of RIPK3 to identify the potential degron motifs for SPOP (Fig. 1A), this study constructed RIPK3 deletion mutants targeting three putative degron motifs in the linker domain (spanning amino acids 287–450). These mutants, designated as ΔDeg-1 (aa 385–389), ΔDeg-2 (aa 399–403), and ΔDeg-3 (aa 409–413) (Supplementary Fig. 2), showed reduced binding to SPOP in the ΔDeg-2 and ΔDeg-3 mutants (Fig. 3C). These results suggest that the RIPK3 degron motif 2 and 3 located in the linker domain play a key role in SPOP binding. The prolonged half-life of RIPK3-ΔDeg-2 and -3 was observed in contrast to that of RIPK3-wt and ΔDeg-1 (Fig. 3D). In particular, the RIPK3-ΔDeg-2 and -3 abrogated SPOP-mediated ubiquitination (Fig. 3E). These results suggested a mode of action for the SPOP and RIPK3 interaction, indicating that a dimer of the Cul3-SPOP E3 ligase complex might recognize and interact with RIPK3 via MATH and RIPK3-Deg2 and -Deg3, which are located in the LK domain (Fig. 3F).

### Identification of phosphor-degron motifs of RIPK3 for SPOP

**Fig. 4:**
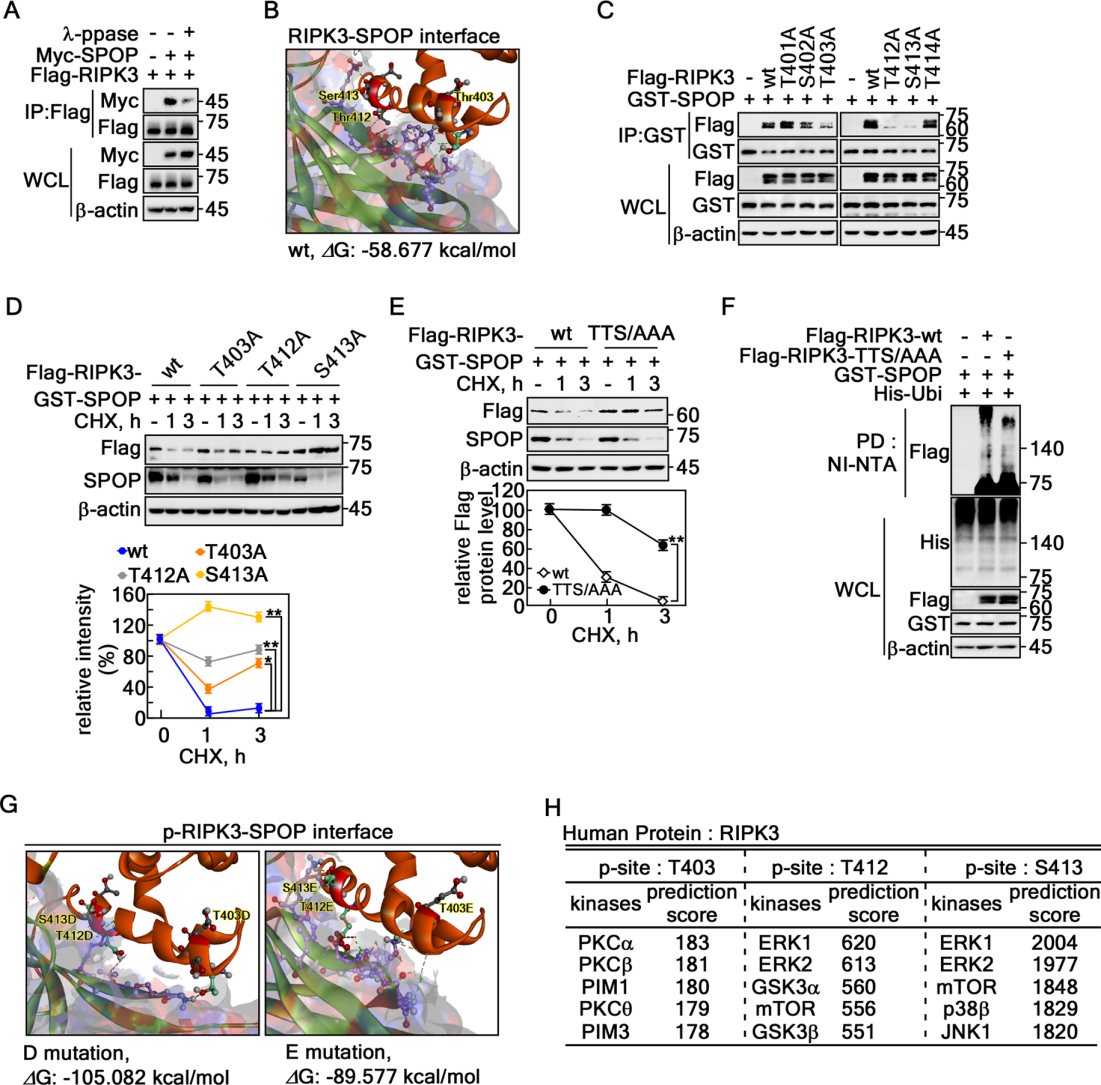
Identification of the phospho-degron motifs of RIPK3 for SPOP. **A** Phosphatase treatment reduced the interaction between SPOP and RIPK3 in HEK293T cells. **B** Structural model showing the interface of protein–protein docking between SPOP and non-phospho-RIPK3 wildtype; ΔG, docking score indicating docking energy. The full model of (B) is provided in Fig. S3A. The interaction types suggested in (B) are summarized in Supplementary Table 1. **C** Reduced interactions between SPOP and each degron motif mutant of RIPK3 were observed in HEK293T cells. **D** Confirmation of the prolonged RIPK3 half-life in degron motif mutants. *Upper panels*: Each of the degron motif mutants shows a prolonged half-life of RIPK3 after the CHX treatment in HeLa cells. *Graphs*: The band intensities of RIPK3 were normalized using the β-actin band intensity. Densitometric computer program: NIH Image J (Ver. 1.42). **E ***Upper panels*: Confirmation of the prolonged half-life of RIPK3 in the triple mutant of RIPK3 at the degron motifs in HeLa cells. wt, wildtype; TTS/AAA, RIPK3-T403A/T412A/S413A. *Graphs*: The band intensities of RIPK3 after normalization using the β-actin band intensity. Densitometric computer program: NIH Image J (Ver. 1.42). **F** Confirmation of the decreased ubiquitination of RIPK3-TTS/AAA in HEK293T cells. **G** Illustration showing the binding interface between SPOP and D or E mutant of RIPK3 (mimetic of phospho-degron motifs of RIPK3). **H** Web-based prediction of kinases to phosphorylate RIPK3 at the degron motifs. **D** and **E** The error bars from three independent experiments indicate the SEM. * *p* < 0.05; ** *p* < 0.01 (Student *t*-test)

Identifying the kinases responsible for phosphorylating RIPK3 at the Deg-2 and Deg-3 sites is crucial, providing critical insights into the mechanism to determine how SPOP regulates RIPK3 stability through phosphorylation, is crucial because SPOP recognizes the phospho-degron of its substrates [31, 36]. The requirement of RIPK3 phosphorylation for the SPOP interaction was proven by IP using cell lysates treated with λ protein phosphatase (λ-ppase) (Fig. 4A). The mode of interaction between SPOP and RIPK3 was analyzed using protein–protein docking via Discovery Studio software. The interaction at RIPK3-Deg-2 and Deg-3 involves 18 hydrogen bonds, seven electrostatic interactions, five hydrophobic interactions, and four combined hydrogen bond-electrostatic interactions, with a binding score of − 58.677 kcal/mol (Fig. 4B, Supplementary Fig. 3A, Supplementary Table 1). These findings provide a basis for constructing RIPK3 point mutants for further study (Supplementary Fig. 3B). The IP result using these constructs showed that Thr403, Thr412, and Ser413 of RIPK3 were involved in the interaction with SPOP (Fig. 4C). The half-life of RIPK3-T403A, -T412A, or -S413A was prolonged compared to the RIPK3-wt control (Fig. 4D). Moreover, the RIPK3-triple mutation at Thr403A, Thr412A, and Ser413A (referred as RIPK3-TTS/AAA, Supplementary Fig. 3B) rescued RIPK3 protein levels (Fig. 4E). In particular, RIPK3-TTS/AAA inhibited SPOP-mediated ubiquitination compared to RIPK3-wt (Fig. 4F). Based on this finding, a protein–protein docking analysis was conducted using RIPK3 with phospho-mimetic amino acid substitutions, replacing the critical residues with aspartic acid (D) or glutamic acid (E). The results suggested that mutating the TTS sequence to DDD or EEE led to a significant decrease in the docking score, from − 58.677 kcal/mol to − 105.082 and − 89.577 kcal/mol, respectively (Fig. 4G, Supplementary Fig. 3C, D). The predicted changes in amino acids from TTS to DDD or EEE suggest that the decreased free energy of binding is likely due to phosphorylation-induced conformational changes (Supplementary Table 1, Supplementary Fig. 3A, C, D). This conformational shift may enhance the stability of the interaction between SPOP and RIPK3 (Supplementary Fig. 3E, F). Putative kinases phosphorylating the Thr403, Thr412, and Ser413 of RIPK3 were predicted by web-based proteomic analysis using a phosphoSite prediction (https://www.phosphosite.org/homeAction) and Group-based prediction system (http://gps.biocuckoo.org/download.php). Based on the prediction scores, Thr403 is likely phosphorylated by PKC α, β, θ, and PIM 1 and 3, while Thr412 and Ser413 may be phosphorylated by mTOR, p38, GSK3 α/β, JNK1, and ERK 1/2 (Fig. 4H). These results showed that phosphorylation of Thr403, Thr412, and Ser413 of RIPK3 plays a key role in interacting with SPOP.

### PIM2-mediated RIPK3 phosphorylation at Thr403 is indispensable for SPOP-mediated RIPK3 ubiquitination

**Fig. 5:**
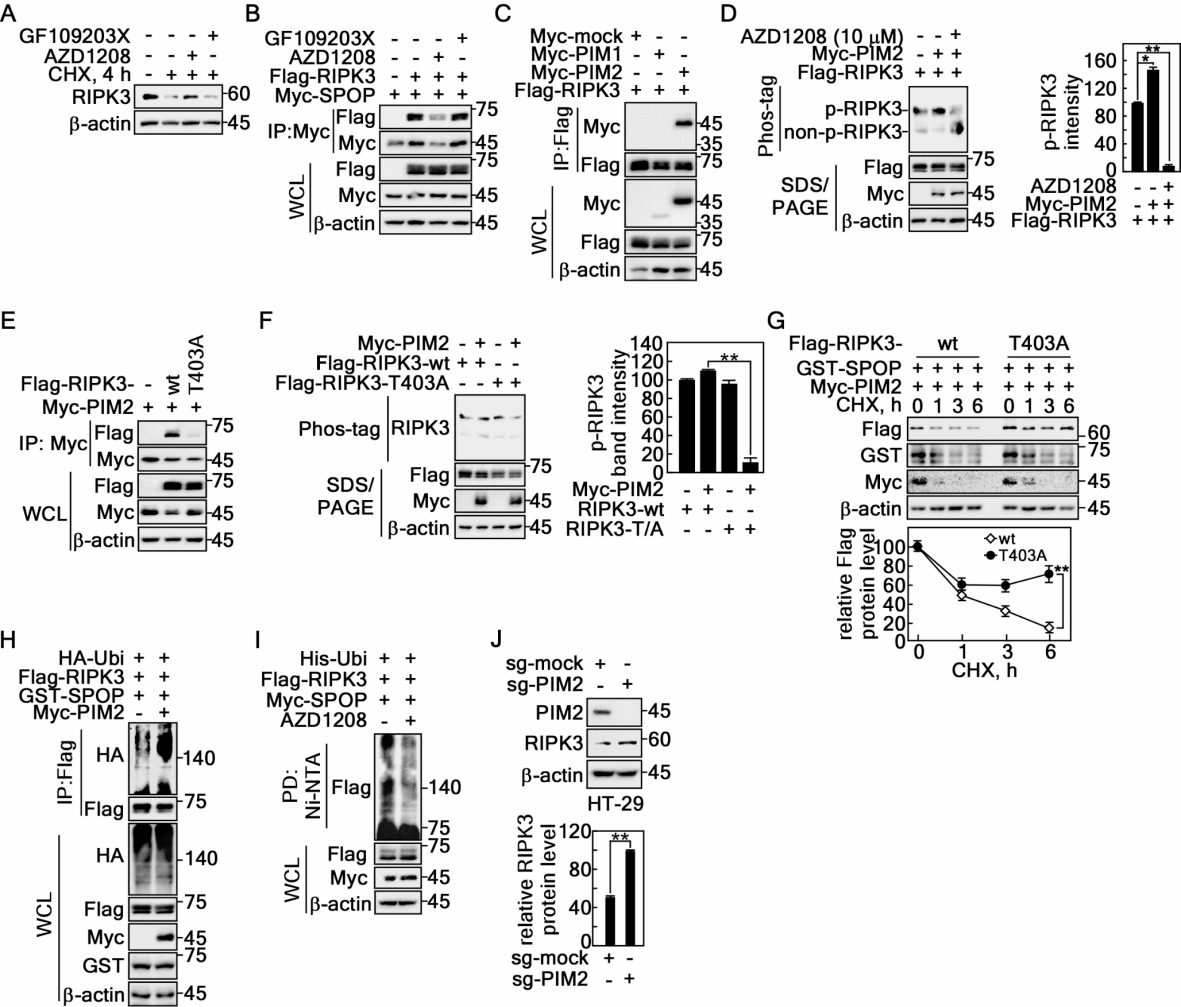
PIM2-mediated RIPK3 phosphorylation at Thr403 induces SPOP-mediated RIPK3 destabilization. **A** PIM inhibition sustained endogenous RIPK3 protein levels, followed by a CHX treatment in HT-29 cells. **B** PIM inhibition abrogated the interaction between SPOP and RIPK3 in HEK293T cells. **C** Confirmation of the specific interaction between RIPK3 and PIM2 detected by IP and western blotting in HEK293T cells. **D** PIM inhibitor abrogated the PIM2-mediated RIPK3 phosphorylation detected by phos-tag gel electrophoresis and western blotting in HEK293T cells. **E** RIPK3 mutation at Thr403 to Ala abrogated the interaction between SPOP and RIPK3 in HEK293T cells. **F** RIPK3 mutation at Thr403 to Ala abrogated PIM2-mediate phosphorylation detected by phos-tag gel electrophoresis and western blotting in HEK-293T cells. **G** SPOP-mediated RIPK3 half-life was prolonged in RIPK3-T403A followed by CHX treatment in HeLa cells. **H** Coexpression of PIM2 with SPOP and RIPK3 enhanced RIPK3 ubiquitination detected by IP and western blotting in HEK293T cells. **I** PIM inhibitor abolished SPOP-mediated RIPK3 ubiquitination detected by the Ni-NTA pulldown assay in HEK293T cells. **J** PIM2 knockout elevated the RIPK3 protein level in sg-PIM2 HT-29 cells. **D**, **F**, **G** and **J** The error bars from three independent experiments indicate the SEM. * *p* < 0.05; ** *p* < 0.01 (Student *t*-test)

The relatively high ERK2 and PIM2 levels in the large intestine, according to the COSMIC data, support the hypothesis that ERK2 and PIM2 may serve as key kinases for phosphorylating RIPK3 at Thr403, Thr412, and Ser413 under physiological conditions (Supplementary Fig. 4A, B). The relationship between RIPK3 phosphorylation at Thr403 and protein destabilization was confirmed by the recovery of the RIPK3 protein level after CHX treatment. AZD1208, a pan-PIM inhibitor, sustained the RIPK3 protein, while GF109203X, a pan-PKC inhibitor, did not (Fig. 5A). At the same time, the RIPK3 and SPOP interaction was abrogated by AZD1208, but not by GF109203X (Fig. 5B). Interestingly, RIPK3 interacts with only PIM2, but not with PIM1 (Fig. 5C). PIM2-mediated RIPK3 phosphorylation was inhibited by the AZD1208 treatment (Fig. 5D). The RIPK3-T403A mutant did not interact with PIM2 (Fig. 5E), resulting in the abrogation of PIM2-mediated RIPK3 phosphorylation (Fig. 5F). The abrogation of the interaction between RIPK3-T403A and PIM2 sustained the RIPK3-T403A protein level after the CHX treatment in contrast to that of RIPK3-wt (Fig. 5G). SPOP-mediated RIPK3 ubiquitination was increased by the coexpression of PIM2 (Fig. 5H). In contrast, AZD1208 inhibited SPOP-mediated RIPK3 ubiquitination (Fig. 5I). As expected, PIM2 knockout by Crispr/Cas9 technology using small guide RNA (sg)-PIM2 increased the RIPK3 protein level (Fig. 5J). These results show that RIPK3 phosphorylation by PIM2 at Thr403 plays a key role in SPOP-mediated RIPK3 ubiquitination.

### RIPK3 phosphorylation by ERK2 at Thr412 and Ser413 is required for SPOP-mediated RIPK3 destabilization

**Fig. 6:**
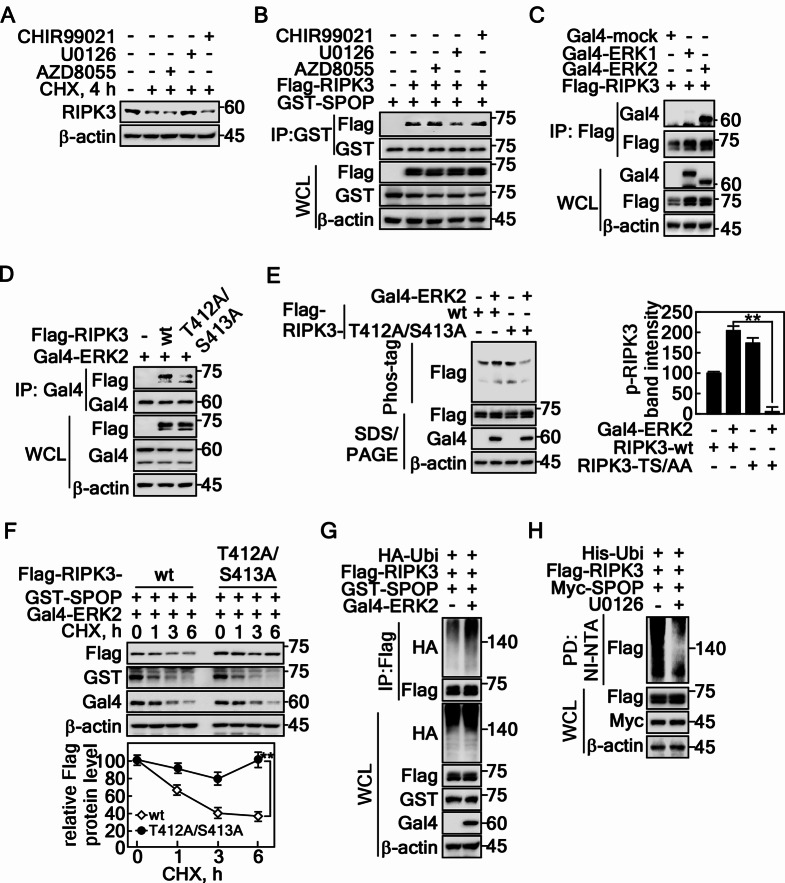
RIPK3 phosphorylation by ERK2 at Thr412 and Ser413 is indispensable for SPOP-mediated RIPK3 degradation. **A** Inhibition of the MEK/ERK signaling pathway sustained endogenous RIPK3 protein levels followed by CHX treatment in HT-29 cells. **B** The inhibition of the MEK/ERK signaling pathway abrogated the interaction between SPOP and RIPK3 detected by IP and western blotting in HEK293T cells. **C** The specific interaction between RIPK3 and ERK2, but not ERK1, detected by IP and western blotting in HEK293T cells. **D** Illustration showing that RIPK3-T412A/S413A reduces the interaction between RIPK3 and ERK2 detected by IP and western blotting in HEK293T cells. **E*** Left panels*: ERK2-mediated RIPK3 phosphorylation was abrogated by mutations at Thr412 and Ser413 of RIPK3 detected by phos-tag gel electrophoresis and western blotting in HEK293T cells. *Graphs*: The band intensities of RIPK3 by normalization using β-actin band intensity. Densitometric computer program: NIH Image J (Ver. 1.42). **F** Confirmation of the prolonged half-life of RIPK3-T412A/S413A followed by CHX (10 µM) treatment in HeLa cells. **G** ERK2 coexpression with RIPK3 enhanced SPOP-mediated RIPK3 ubiquitination by IP and western blotting in HEK293T cells. **H** Chemical inhibition of ERK activity decreased SPOP-mediated RIPK3 ubiquitination detected by NI-NTA pulldown assay in HEK293T cells. **A-H** β-actin was used as an internal control for an equal protein loading. **E** and **F** The error bars from three independent experiments indicate the SEM. ** *p* < 0.01 (Student *t*-test)

Based on the findings from Fig. 4H, this study examined the effects of chemical inhibitors such as CHIR99021 (GSK3), AZD8055 (mTOR), and U0126 (MEK/ERK) on the RIPK3 protein levels. U0126, which inhibits the MEK/ERK pathway, sustained the RIPK3 levels (Fig. 6A). In addition, U0126, but not CHIR99021 or AZD8055, disrupted the interaction between RIPK3 and SPOP (Fig. 6B), suggesting that ERK-mediated RIPK3 phosphorylation is crucial for SPOP binding. This was confirmed as RIPK3 interacted specifically with ERK2 but not ERK1 (Fig. 6C). Hence, ERK2 might act as a kinase to phosphorylate RIPK3. In particular, the ERK2 and RIPK3 interaction was decreased by the mutation of RIPK3 at T412 and S413 to Ala (Fig. 6D, Supplementary Fig. 3B), resulting in the abrogation of ERK2-mediated RIPK3 phosphorylation (Fig. 6E). The reduced interaction and phosphorylation of RIPK3-T412A/S413A with ERK2 resulted in sustained RIPK3 protein levels in contrast to that of RIPK3-wt (Fig. 6F). SPOP-mediated RIPK3 ubiquitination was increased by the coexpression of ERK2 compared to that of the mock control (Fig. 6G). In contrast, SPOP-mediated RIPK3 ubiquitination was suppressed by the U0126 treatment (Fig. 6H), suggesting that SPOP-mediated RIPK3 ubiquitination is required the ERK2-mediated RIPK3 phosphorylation. These results clearly show that PIM2 and ERK2 are crucial kinases to regulate SPOP-mediated RIPK3 ubiquitination and destabilization, as shown in Fig. 5.

### SPOP depletion sensitizes RIPK3-mediated necroptotic cell death by lipopolysaccharide (LPS)/sMAC/zVAD stimulation

**Fig. 7:**
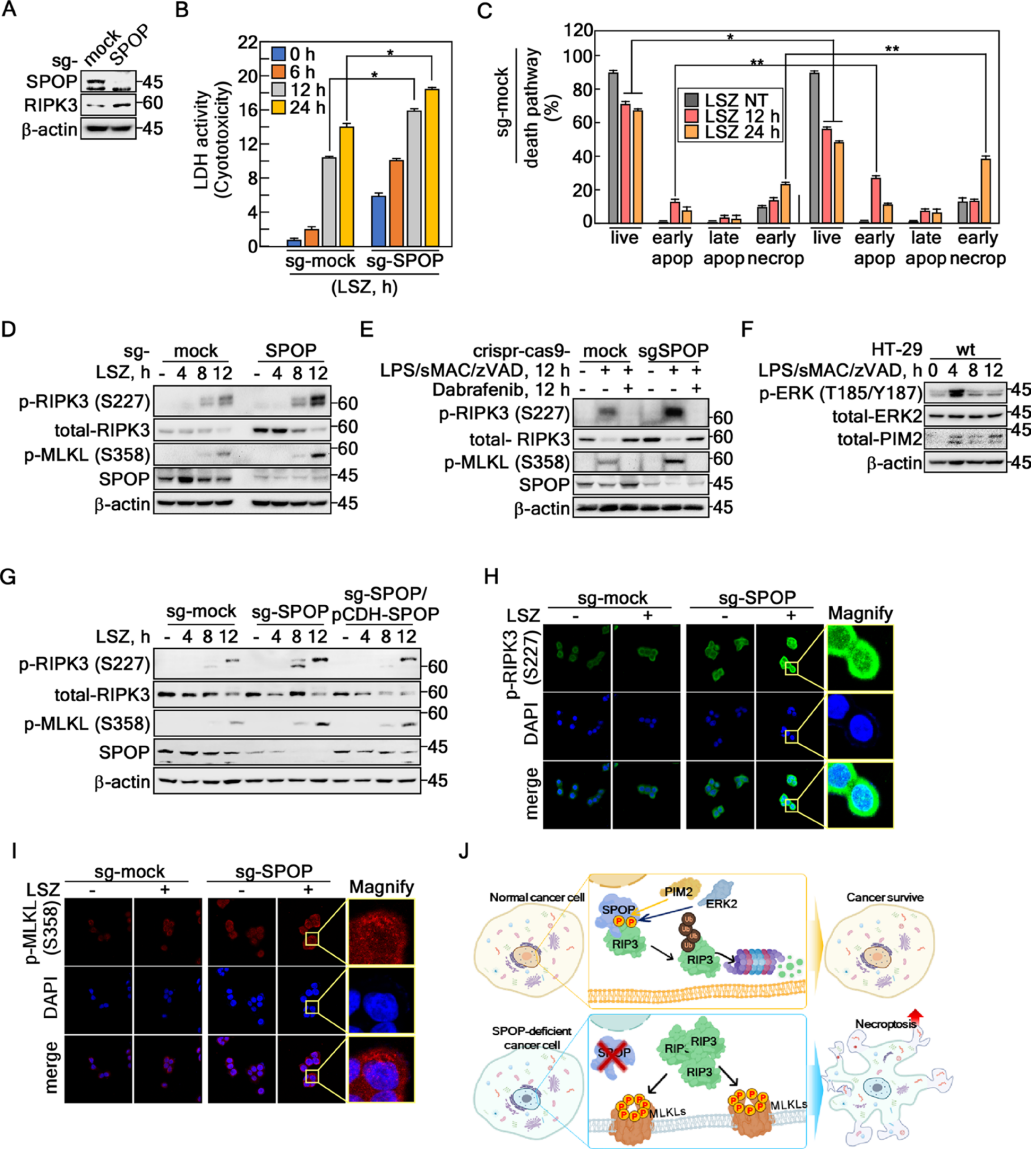
SPOP knockout sensitizes RIPK3-mediated necroptosis. **A** Confirmation of increased RIPK3 protein levels by SPOP knockout using small guide RNA-SPOP (sg-SPOP) in HT-29 cells. **B** Confirmation of the increased LSZ-induced cell toxicity of SPOP knockout cells by LDH activity analysis in HT-29 cells. **C** Death pathway tracking of LSZ-treated SPOP knockout cells using Annexin V and propidium Iodide staining by flow cytometry in HT-29 cells. **D** RIPK3 and MLKL phosphorylation was increased by the LSZ treatment in SPOP knockout HT-29 cells. **E** The activation of SPOP-RIPK3-mediated necroptosis signal molecules was abrogated by the Debrafenib (10 µM, 12 h) treatment. **F** Confirmation of the change in protein level of phospho-ERKs and total-PIM2 under the LSZ treatment at the indicated times. **G** Increased phospho-RIPK3 and -MLKL protein levels by LSZ treatment in sg-SPOP HT-29 cells abrogated by SPOP re-introduction into sg-SPOP HT-29 cells. **H** and **I** Illustrations showing that SPOP knockout increased LSZ-induced phospho-RIPK3 at Ser227 (**H**, ×400) and phospho-MLKL at Ser358 (**I**, ×400) by ICF under a confocal microscope in HT-29 cells. **J** The graphic abstract for this research indicates that SPOP acts as an E3 ligase for RIPK3, inhibiting necroptotic cell death in normal cancer cells. On the other hand, when the SPOP activity was depleted, increased RIPK3 protein accelerated necroptotic cell death when the cancer cells were stimulated with necroptosis inducers. **A** and **D-G** β-actin was used as an internal control for an equal protein loading. **B** and **C** The error bars from a triplicate experiment indicate SEM. * *p* < 0.05; ** *p* < 0.01 (Student *t*-test)

SPOP-depleted HT-29 cells were generated using CRISPR/Cas9 with small guide RNA (sg-SPOP) to investigate the biological impact of SPOP-mediated regulation of RIPK3 stability (Fig. 7A, Supplementary Fig. 5A). The RIPK3 protein levels were higher in the SPOP-depleted cells than the sg-mock control cells (Fig. 7A). Contrary to expectations, SPOP-depleted cells exhibited similar proliferation rates to sg-mock cells under normal, unstressed culture conditions (Supplementary Fig. 5B). On the other hand, the cells exhibited severe morphological changes when SPOP-depleted cells were stimulated with lipopolysaccharide (LPS)/sMAC/zVAD (referred as LSZ), a necroptosis inducer that inhibits caspase-8 and cIAP. These changes included floating, expansion, and ghost formation at the early time point compared to sg-mock control cells (Supplementary Fig. 5C). The LDH cytotoxicity assay showed that SPOP depletion increased the cytotoxicity for LSZ stimulation compared to that of the sg-mock cells (Fig. 7B). Tracking of the cell death route by flow cytometry using propidium iodide and Annexin V indicated that SPOP knockout increased the cell population of early apoptosis and early necroptosis. The live cell population was decreased sharply by the LSZ treatment compared to sg-mock control cells (Fig. 7C, Supplementary Fig. 5D), indicating that SPOP depletion sensitizes necroptotic cell death to LSZ stimulation. The increased necroptosis sensitivity in the SPOP-depleted cells was proven by the increase of RIPK3 and MLKL phosphorylation by LSZ treatment (Fig. 7E). Importantly, Dabrafenib, known as a RIPK3 kinase-dependent necroptosis inhibitor [37–39], abolished the LSZ-induced p-RIPK3-Ser227 and p-MLKL-Ser358 (Fig. 7E), showing that SPOP-mediated increased cytotoxicity by an LSZ treatment is caused by necroptosis (Fig. 7E). Interestingly, the LSZ treatment increased phosphorylation of RIPK3 at Ser227 and MLKL at Ser358 at 12 h in sg-mock and SPOP-depleted cells. Furthermore, phosphorylated ERK and the total PIM2 levels were elevated at 4 h but declined by 8 h after the LSZ treatment in HT-29 cells (Fig. 7F). These findings suggest that the ERK- and PIM2-mediated cell survival pathways were activated in the early phase, while the necroptotic death pathway was triggered in the later response, indicating the potential decoupling of these two processes. Moreover, SPOP re-introduction into sg-SPOP cells (sg-SPOP/pCDH-SPOP) restored the phosphor-protein levels of RIPK3 and MLKL at a level similar to the sg-mock cells (Fig. 7G). Importantly, ICF analyses using SPOP-depleted HT-29 cells showed a strong increase in phospho-RIPK3 at Ser277 (Fig. 7H) and phospho-MLKL at Ser358 (Fig. 7I) after the LSZ treatment compared to that of the sg-mock-LSZ-treated cells. Overall, these results showed that the SPOP-mediated RIPK3 stability plays a key role in regulating RIPK3-dependent necroptotic cell death (Fig. 7J).

## Discussion

SPOP is classified as a tumor suppressor based on extensive studies, including those on cell proliferation, migration, and invasion. On the other hand, several studies have shown that an SPOP deficiency did not significantly affect cell proliferation according to an MTS and CCK-8 assay [40, 41], even though SPOP depletion increases cell migration and invasion in cancer [40]. These results suggest that SPOP-mediated cellular phenotypes, particularly in cell proliferation, may vary across cancer types depending on the progression stage and genotype of that cancer. Previously, ELK3 was reported to be a substrate of SPOP and plays a crucial role in regulating the expression of the *c-fos* gene. c-Fos, a key transcription factor, controls the expression of more than 50–60% of intracellular proteins by forming a dimer with c-Jun, which are recognized as proto-oncogenes [42]. In addition, many proteins identified as substrates of SPOP are involved in cancer development and progression, classifying SPOP as a tumor suppressor. This correlation explains why low levels of SPOP protein are frequently observed in cancer cells. This study elucidated the role of the SPOP-RIPK3 signaling pathway in necroptotic cell death. From this viewpoint, the ERKs and PIM2 signaling pathways, which play a key role in cell survival and cancer malignancy, may lead to cancer cell resistance to necroptosis by suppressing SPOP. Interestingly, the destabilization of RIPK3 mediated by SPOP might be enhanced because the SPOP and RIPK3 protein levels show an inverse correlation. In this context, the ERK2 and PIM2-mediated phosphorylation of RIPK3 may promote its ubiquitination and degradation, even at low SPOP levels. This increased destabilization of RIPK3 could prevent necroptotic cell death when apoptosis is suppressed, leading to chemoresistance against anti-cancer drugs. The ERK2 and PIM2-mediated phosphorylation of RIPK3 reduces its stability, decreasing the necroptosis sensitivity and contributing to overall chemoresistance. Therefore, a mechanism whereby SPOP-mediated RIPK3 regulation may sensitize cancer cells to death through SPOP modulation was proposed (Fig. 7J).

When cells undergo necroptosis, they release endogenous molecules, such as subcellular organelles, ribosomes, and nucleic acids (DNA from the nucleus and RNA), into the extracellular space as the dying cells disintegrate. These released components act as damage-associated molecular patterns (DAMPs), triggering inflammation and an immune response in the neighboring cells [43]. The released endogenous molecules are known as damage-associated molecular patterns (DAMPs), including the extended IL-1 family (IL-1α, IL-1β, IL-18, IL-33, and IL-36α, β, and γ) [44]. These DAMPs leakage by cytoplasmic membrane rupture can activate inflammasomes, increase the recruitment of inflammatory cells to the infection site, and facilitate and promote subsequent virus-specific T-cell responses to induce inflammation [45]. SPOP knockout by sg-SPOP might affect the subcellular organization of components involved in RIPK3-mediated necroptosis because SPOP downregulated the RIPK3 protein levels. Precise regulation of necroptosis might serve as an alternative cell death program to prevent cancer because necroptosis is a critical cell-killing mechanism in response to severe stress and blocked apoptosis [46]. Many scientists consider RIPK3 primarily a cytoplasmic protein because of its role in necroptosis. On the other hand, recent studies have shown that RIPK3 shuttles between the cytosol and the nucleus, facilitated by a nuclear localization signal located between amino acids 442–472 [21, 47]. In addition, although the phosphorylated RIPK3 is mainly localized to the cytosol, a considerable amount of phosphorylated RIPK3 at Ser227 was also observed in the nucleus (Fig. 7H). Cytosolic phospho-RIPK3 proteins are mainly oligomerized form to induce necroptosis because phospho-RIPK3-Ser227 was increased mainly at the cytoplasm by Crispr/Cas9-mediated SPOP depletion when the cells were stimulated by LSZ (Fig. 7D, E, G), resulting in an increase in necroptotic cell death by LSZ (Fig. 7B, C). Hence, it is plausible that regulation of the cytosolic RIPK3 content occurs within the nucleus because nuclear RIPK3 remains active and leads to MLKL activation in the nucleus. Accordingly, SPOP-mediated RIPK3 destabilization could be an essential mechanism for controlling the amount of cytosolic phospho-RIPK3 protein.

These findings suggest that RIPK3 is phosphorylated by ERK2 and PIM2, which can localize to the nucleus upon activation. For example, active ERKs translocate to the nucleus through interaction with importin 7 following the phosphorylation of their SPS motif. PIM2, a short-lived protein primarily regulated by protein stability, is present in the cytoplasm and nucleus. Thus, the ERK2- and PIM2-mediated phosphorylation of RIPK3 likely occurs in the nucleus. Given that SPOP is generally nuclear, this phosphorylation may facilitate RIPK3 destabilization through SPOP-mediated ubiquitination, reducing cytoplasmic oligomerization. SPOP facilitated the K48 ubiquitination of RIPK3 (Fig. 2K). Therefore, this paper proposes that SPOP-mediated RIPK3 ubiquitination suppressed RIPK3-mediated cell death. This notion was supported by SPOP depletion increasing the LDH activity in the culture medium by an LSZ treatment (Fig. 7B), indicating that necroptosis by cell membrane rupture was induced. Consequently, the ERK2- or PIM2-SPOP-RIPK3 signaling pathway might confer resistance to necroptosis. Although this study did not establish in vivo relevance for this pathway, future research involving combined treatment with SPOP inhibitors and necroptosis-inducing stimuli or anti-cancer drugs might enhance anti-cancer efficacy in chemoresistant cancer cells in in vivo models. Furthermore, COSMIC data analysis suggested that cancers in the large intestine have relatively high levels of PIM2 and ERK2. Clinical studies showed that SPOP mutations are present in up to 15% of prostate cancer cases [48, 49]. Mutations in SPOP are found primarily in the MATH domain [50], which accelerates cancer progression by promoting the accumulation of oncogenic substrates, leading to increased cell proliferation, migration, and invasion [31, 51]. Although the role of SPOP in colorectal cancers is unclear, SPOP downregulation is observed in approximately 20–61% of colorectal cancer patients despite the rarity of SPOP mutations in colorectal cancer [31, 50, 51]. Therefore, studying the efficacy of the ERK2/PIM2-SPOP-RIPK3 signaling pathway in chemoresistance mechanisms could be pivotal for developing broader cancer treatment strategies.

Regarding the regulation of cancer cell necroptosis by SPOP, this study suggests that PIM2 and ERK2 kinase can fine-tune key molecules through phosphorylating T403, T412, and S413 of RIPK3 (Figs. 5 and 6). Based on the structural model of SPOP-CUL3-Rbx1, SPOP does not operate as a monomer to exhibit functional activity but as a dimer. Moreover, the oligomerized SPOP complex facilitates the recognition and binding of degron motifs on the substrate [52, 53]. Given these structural characteristics, this paper proposed an interaction model indicating that one POP molecule may bind to the p-Thr403 residue of the degron motif of RIPK3 and another to the p-Thr412 and p-Ser413 residues of the degron motif of RIPK3. This study provides a monomer binding model based on the molecular biological data obtained through IP for the degron motif deletion and point mutation mutants of RIPK3 against SPOP (Figs. 3 and 4). Importantly, the binding score and interaction stabilization score simulated by protein-protein docking showed that the amino acid replacement at Thr403, Thr412, and Ser413 to negatively charged amino acids, such as Asp or Glu, reduced the binding energy from − 58 kcal/mol to − 105 and − 89 kcal/mol, respectively (Fig. 4B vs. G). Thus, the SPOP-mediated regulation of RIPK3 may play a critical role in various human diseases associated with inflammation and immune responses.

Necroptosis is a form of cell death characterized by increased phosphorylation levels of RIPK3 and mixed lineage kinase domain-like protein (MLKL). Although tumor necrosis factor-α (TNF-α)-mediated necroptosis is used widely as a stimulus, recent studies suggest that the phosphorylation of MLKL can also be induced by the ligand of Toll-like receptor 4, lipopolysaccharide (LPS) [54, 55]. Moreover, the use of LPS to induce necroptosis in this study is supported by evidence that necroptosis can be induced by various stimuli, including TNF, Fas, TNF-related apoptosis-inducing ligand (TRAIL), and Toll-like receptor (TLR) ligands, such as LPS, via the inhibition of the caspase and cellular inhibitor of apoptosis protein (cIAP) [43]. Furthermore, in potency for necroptosis, LPS-induced necroptosis may be more potent than TNF-α-induced necroptosis [54]. Therefore, this study used a strategy of inducing necroptosis using LPS instead of TNF-α.

In cell fate regulation, PIMs and ERKs are crucial for promoting cell survival and proliferation [16, 56–58]. The PIM2- and ERK2-mediated phosphorylation of RIPK3 at its degron motifs may be a key signaling pathway determining whether cancer cells survive or undergo necroptotic death. SPOP is an E3 ligase recognizing phospho-degrons. Hence, its interaction with RIPK3 is essential for RIPK3 destabilization. A disruption of RIPK3 phosphorylation, either chemically or genetically, inhibits this interaction, establishing the PIM2/ERK2-RIPK3-SPOP axis, which enhances necroptosis sensitivity. On the other hand, although SPOP knockout increased the RIPK3 protein levels (Fig. 7A), this elevation alone did not affect cell proliferation, as shown by the similar growth rates observed between SPOP-depleted and the control cells (Supplementary Fig. 5B). Accordingly, while SPOP regulates the RIPK3 stability, additional factors or conditions may be required for RIPK3 to influence cell proliferation. Therefore, when cells are stimulated with TNF, the ERK and PIM signaling pathways play a crucial role in determining the route of regulated cell death. These pathways help dictate whether the cells undergo apoptosis, necroptosis, or activate NF-κB-mediated survival mechanisms [59–61]. Moreover, these kinases are important targets for developing specific inhibitors against human diseases, including cancers and chemoresistance. Therefore, modulating the SPOP-RIPK3 signaling pathway with such inhibitors may offer an alternative strategy to control cell proliferation, inflammation, apoptosis, and necroptosis in cancer cells. SPOP depletion did not affect cell proliferation under normal culture conditions without external stimuli. Therefore, it may be feasible to use a localized delivery of specific inhibitors to influence cancer cell fate. This approach could modulate the SPOP-RIPK3 pathway, allowing for the targeted control of processes, such as apoptosis, necroptosis, and inflammation, without affecting normal cell proliferation.

## Electronic supplementary material

Below is the link to the electronic supplementary material.

Supplementary Material 1

Supplementary Material 2

Supplementary Material 3

Supplementary Material 4

## Data Availability

The source data and any additional information in this paper will be shared upon reasonable request.
