# Supplementary material for: SPOP-mediated RIPK3 destabilization desensitizes LPS/sMAC/zVAD-induced necroptotic cell death
Source: Cell Mol Life Sci. 2024 Nov 14;81(1):451. doi: 10.1007/s00018-024-05487-7 (PMC11564579; doi:10.1007/s00018-024-05487-7)
Supplement: Supplementary file 1 — Supplementary Material 1 [file 18_2024_5487_MOESM1_ESM.pdf]

[Supplementary information]

SPOP-mediated RIPK3 destabilization desensitizes LPS/sMAC/zVAD-induced necroptotic cell death

Ga-Eun Lee[1,2], Geul Bang[3], Jiin Byun[1], Weidong Chen[1], Dohyun Jeung[1], Hana Cho[4], Joo Young Lee[1,5], Han Chang Kang[3,5], Hye Suk Lee[1,5], Jin Young Kim[3], Kwang Dong Kim[6], Wu Juan[1], Soo Bin Nam[2], Young Jik Kwon[7], Cheol-Jung Lee[2,*], and Yong-Yeon Cho[1,5,*]

Contents:

1. One Supplementary Table**: Supplementary Table 1**
2. Five Supplementary Figures**: Supplementary Fig. 1-5**

Supplementary Table 1. Comparison of binding energy and interaction amino acid residues involved in SPOP and RIPK3 interaction. For ZDock, wildtype, D, and E mutants of RIPK3 at putative degron motifs of RIPK3 were used for the ZDock.

ZDock Score between RIPK3 and SPOP				ZDock Score between p-RIPK3 and SPOP					
ZRank Score	-58.677 kcal/mol			-105.082 kcal/mol			-89.577 kcal/mol		
Interaction type	amino acid RIPK3	amino acid SPOP	Distance (Å)	amino acid RIPK3	amino acid SPOP	Distance (Å)	amino acid RIPK3	amino acid SPOP	Distance (Å)
Hydrogen bond	Gly249	Asp77	2.31	Asn424	Met35	2.19	Gln247	Met48	2.49
	Thr401	Gln127	2.99	Thr401	Gly26	2.11	Gly249	Asp77	2.03
	Gln425	Ala116	2.32	Pro411	Lys31	3.03	Thr401	Gln127	2.02
	Leu245	Lys81	2.53	Asp412	Lys31	2.04	Arg405	Gly128	2.43
	Ala248	Lys81	3.06	Pro411	Lys31	2.75	Glu244	Lys74	2.64
	Gly249	Lys81	2.91	Asn424	Met35	2.02	Gly249	Lys81	2.87
	Asn424	Lys115	2.43	Asp413	Lys101	2.34	Glu413	Tyr87	3.08
	Gln425	Glu118	2.22	Pro409	Lys101	2.50	Thr401	Gln127	3.09
	Ser389	Arg121	2.22	Trp348	Lys115	2.80	Phe404	Lys129	1.84
	Thr387	Arg121	2.21	Glu244	Lys134	2.81	Asp390	Lsy129	1.98
	Asp390	Lys129	2.75	Arg236	Glu79	3.42	Glu412	Gly132	2.13
	Phe404	Lys129	1.84	Pro421	Glu113	3.79	Glu413	Lys134	1.97
	Asp390	Trp131	2.96				Gly249	Ser80	3.73
	Thr416	Lys134	2.10				Asp390	Lys129	3.02
	Ser413	Lys134	2.63						
	Gly249	Ser80	2.98						
	Lys364	Asp77	3.14						
	Thr412	Gly132	3.48						
Electrostatic interaction	Lys282	Asp77	5.29	Arg236	Glu79	3.49	Lys364	Asp77	5.01
	Lys282	Glu78	2.95	Arg239	Glu79	4.72	Arg405	Asp130	4.07
	Lys364	Glu78	4.59	Glu251	Lys115	4.86	Glu244	Lys74	4.31
	Arg405	Asp130	5.50	Glu347	Arg138	5.20	Asp390	Lys129	3.59
	Glu244	Lys74	4.64				Phe404	Lys129	4.43
	Gln425	Lys103	4.99						
	Phe404	Lys129	3.87						
Hydrophobic interaction	Ala248	Met48	3.35	Pro409	Val29	5.26	Ala248	Met48	3.48
	Ala248	Lys81	3.97	Pro409	Val164	5.44	Ala248	Lys81	3.96
	Arg405	Lys129	4.89	Pro421	Leu107	4.63	Arg405	Lys129	4.36
	Met408	Lys129	5.13	Lys351	Ala116	4.46	Met408	Lys129	5.09
	Phe404	Val126	4.99	Val343	Ala146	4.76	Phe404	Val126	5.09
				Pro421	Phe158	4.52	Met408	Trp131	4.08
Hydrogen bond : Electrostatic interaction	Lys351	Glu79	2.19				Lys282	Glu78	2.05
	Lys364	Asp77	3.28				Lys351	Glu79	2.85
	Glu251	Lys81	1.95				Glu251	Lys81	2.07
	Asp390	Lys129	1.73						
Other	Met408	Asp130	3.27	Arg422	Met35	3.06	Met408	Trp131	2.65
	Met408	Trp131	3.25						

Supplementary Table 1 Detailed information on SPOP and RIPK3 docking. The blue letters indicate the degron motifs suggested in **Fig. 3C**.

Suppl Fig. 1 A+B+C+D+E by GE Lee

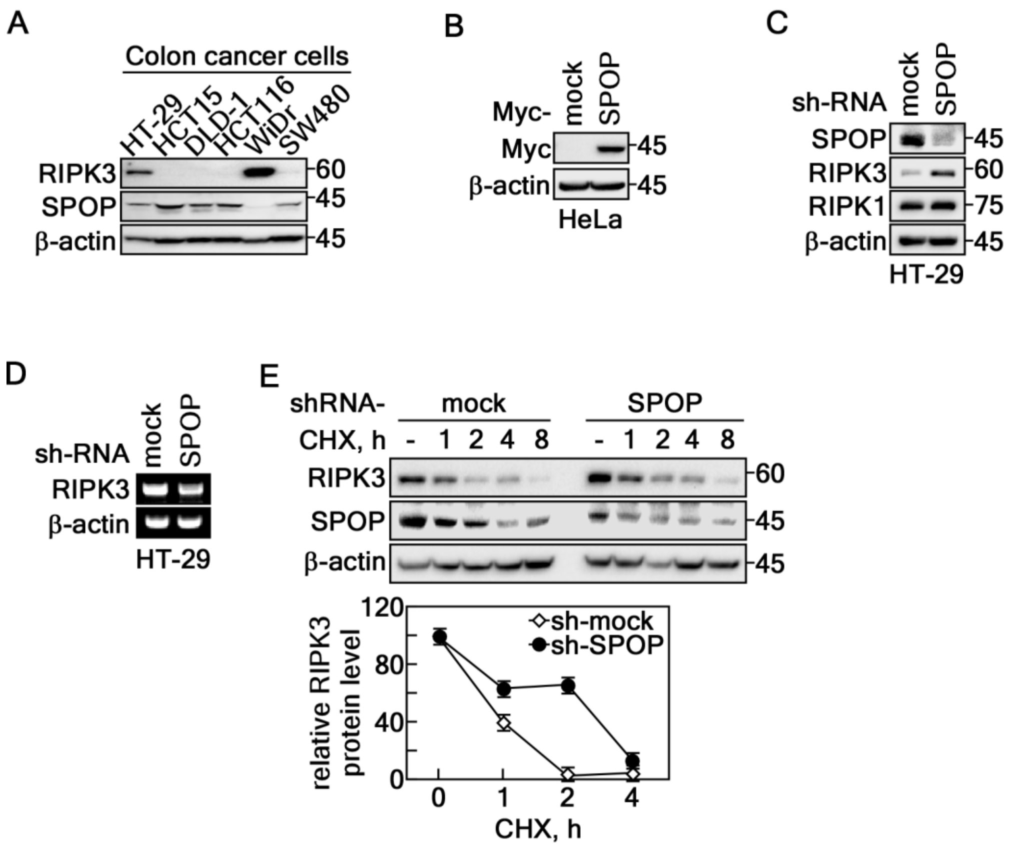

Supplementary Fig. 1 SPOP regulates the RIPK3 half-life. **A** Confirmation of the RIPK3 and SPOP protein levels in colon cancer cells, such as HT-29, HCT15, DLD-1, HCT116, WiDr, and SW480. **B** Confirmation of SPOP overexpression in HeLa cells. **C** SPOP knockdown by sh-SPOP increased RIPK3, but not RIPK1, in HT-29 cells. **D** SPOP knockdown-mediated RIPK3 reduction was not caused by RIPK3 gene expression in HT-29 cells. **E** Measurement of the prolonged RIPK3 half-life by SPOP knockdown HT-29 cells. The error bars from a triplicate experiment indicate SEM.

Suppl Fig. 2 by GE Lee

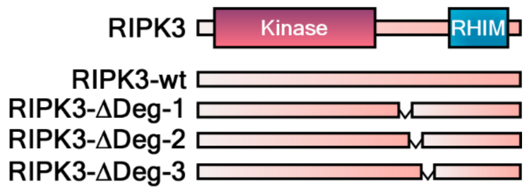

Supplementary Fig. 2 Illustration showing the maps of SPOP and RIPK3. Schematic maps for degron motif deletion mutants of RIPK3. ΔDeg-1, deleted aa 385-389; ΔDeg-2, deleted aa 399-403; and ΔDeg-3, deleted aa 410-414.

Suppl Fig. 3 A+B+C+D+E+F by GE Lee

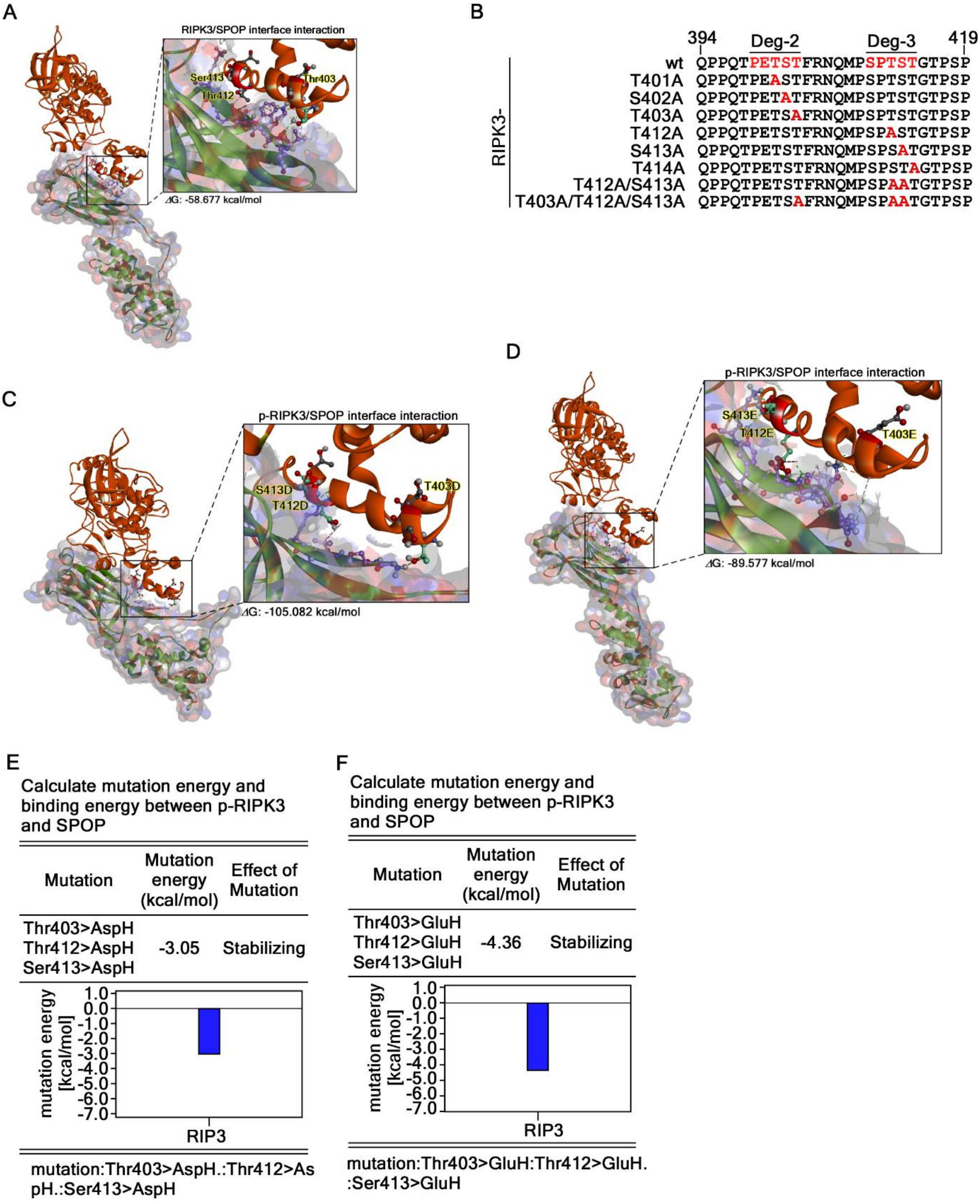

Supplementary Fig. 3 Phospho-mimetic mutations of RIPK3 at the degron motifs affect complex stability. **A** Full picture of the docking interface between SPOP and RIPK3-wt. The magnified boxed area is shown in **Fig. 4B**. **B** Mutation strategy of RIPK3 at Deg-2 and Deg-3. **C-D** Full pictures of the

docking interface between SPOP and RIPK3-TTS/DDD or -TTS/EEE. The magnified boxed area is shown in **Fig. 4G**. **E-F** Valuation of the binding effect for the complex stabilization between SPOP and RIPK3-TTS/DDD or -TTS/EEE.

Suppl Fig. 4 A+B by GE Lee

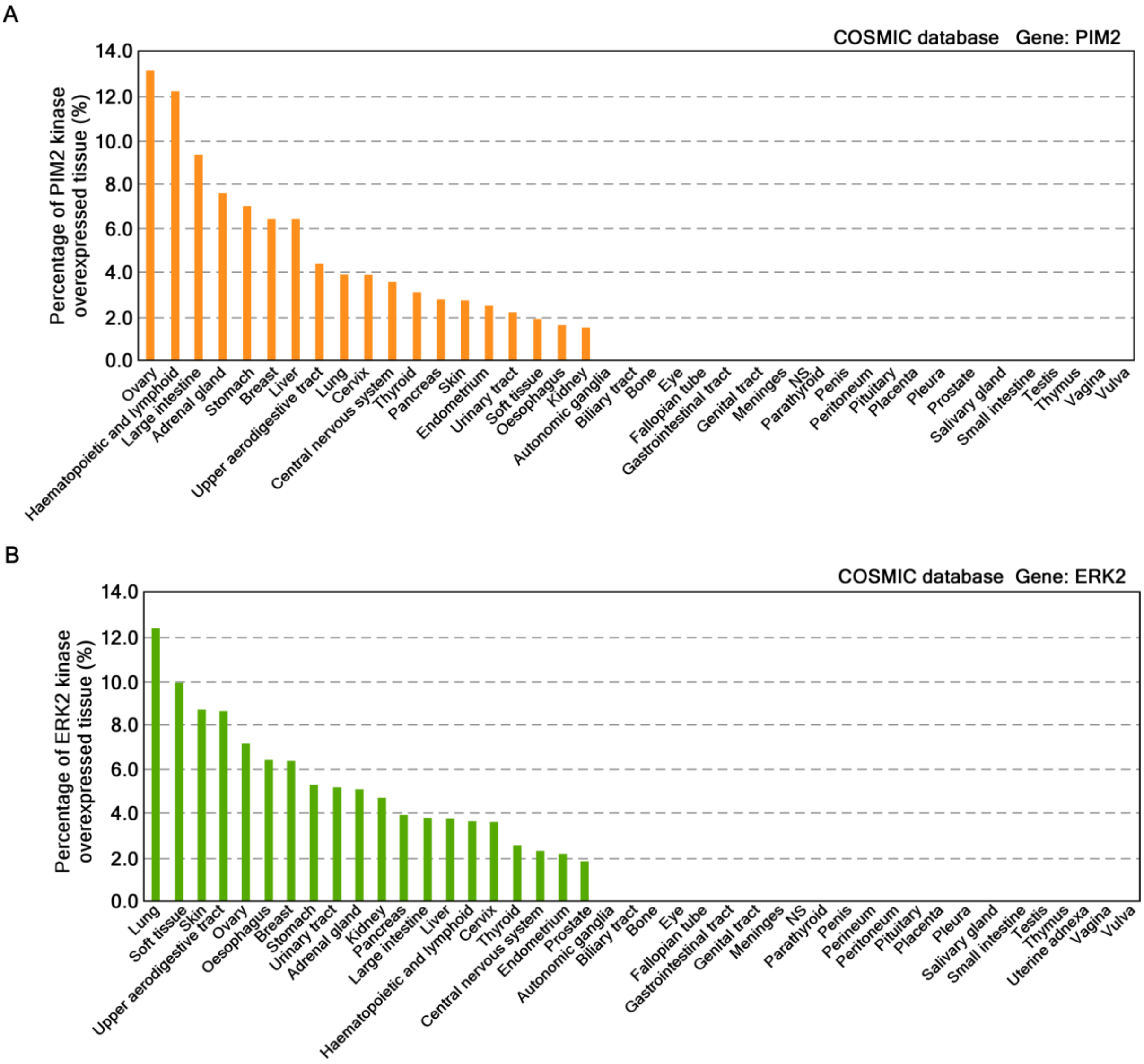

Supplementary Fig. 4 Comparison of the ERK2 and PIM2 protein levels in cancer tissues. **A-B** COSMIC database was used to analyze the expression levels of ERK2 (**A**) and PIM2 (**B**) in various human cancer tissues.

Suppl Fig. 5 A+B+C+D by GE Lee

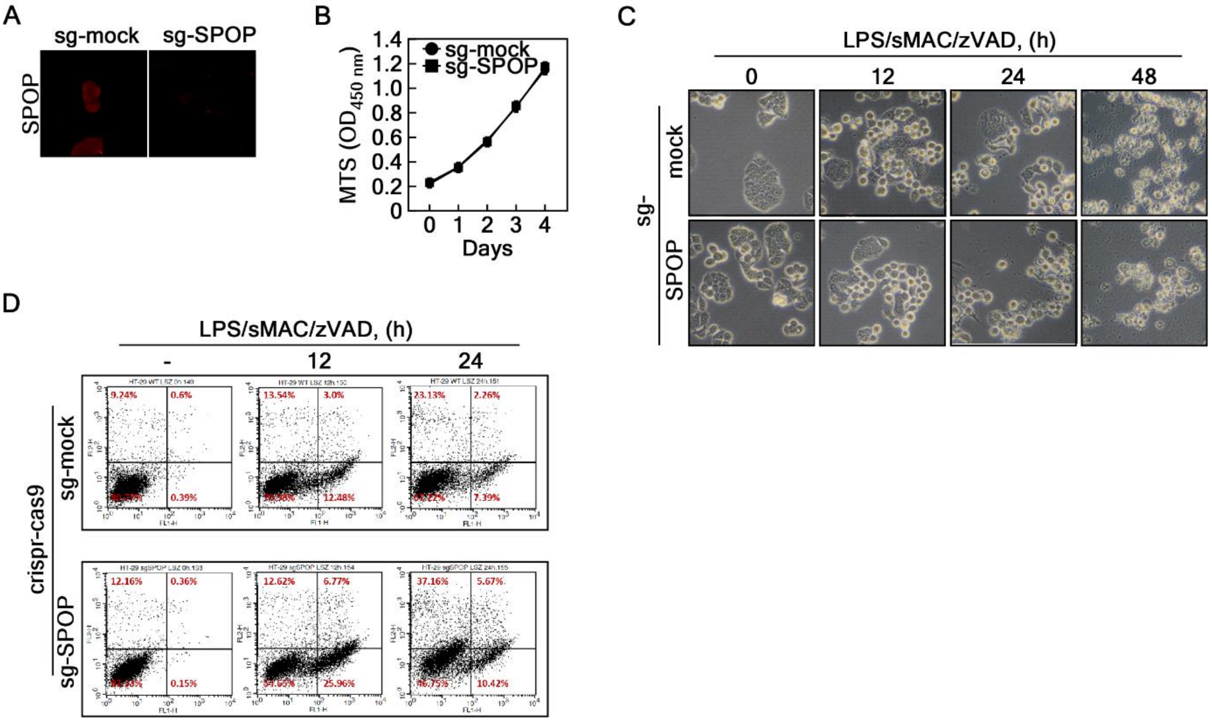

Supplementary Fig. 5 SPOP knockout sensitized LSZ-induced necroptosis. **A** Illustration confirming SPOP knockout using sg-SPOP by ICF analysis under a confocal microscope in HT-29 cells. **B** SPOP knockout did not affect cell proliferation under normal cell culture conditions analyzed by the MTS assay in HT-29 cells. **C** SPOP knockout sensitized LSZ-induced necroptotic cell death morphology observed by phase-contrast optical microscopy (×200) in HT-29 cells. **D** Genetic depletion of SPOP-sensitized LSZ-induced cell death measured by flow cytometry in HT-29 cells. The measured cell populations were divided into AV$^-$/PI$^-$ (alive cells), AV$^+$/PI$^-$ (early apoptotic cells), AV$^-$/PI$^+$ (early necrosis/necroptosis cells), and AV$^+$/PI$^+$ (late apoptosis and necrosis/necroptosis cells). These supplementary data support **Fig. 7C**.
