# Supplementary material for: SPOP-mediated RIPK3 destabilization desensitizes LPS/sMAC/zVAD-induced necroptotic cell death
Source: Cell Mol Life Sci. 2024 Nov 14;81(1):451. doi: 10.1007/s00018-024-05487-7 (PMC11564579; doi:10.1007/s00018-024-05487-7)

Whole blots Fig. 1 by Lee

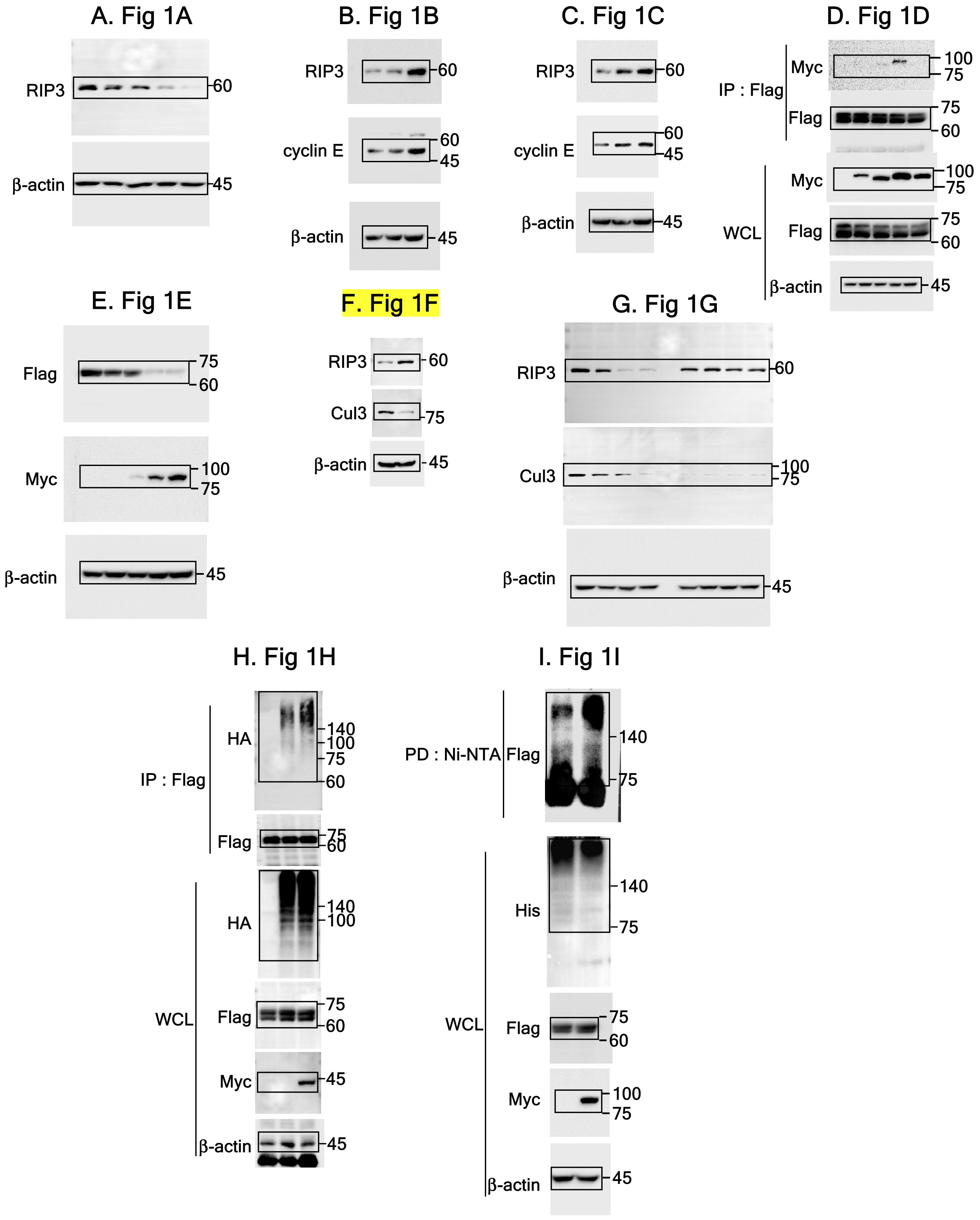

Whole blots Fig. 2 by Lee

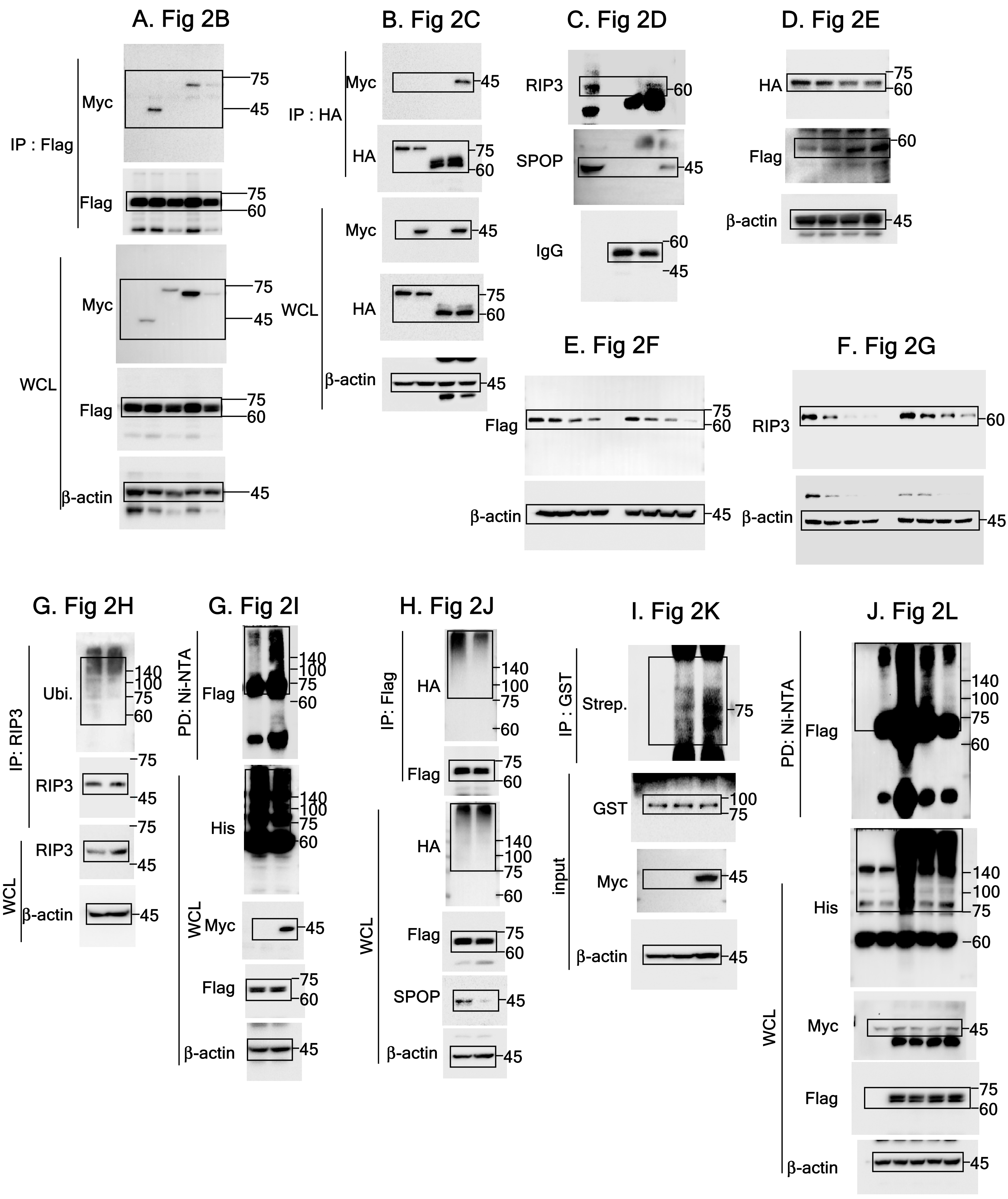

A. Fig 3B

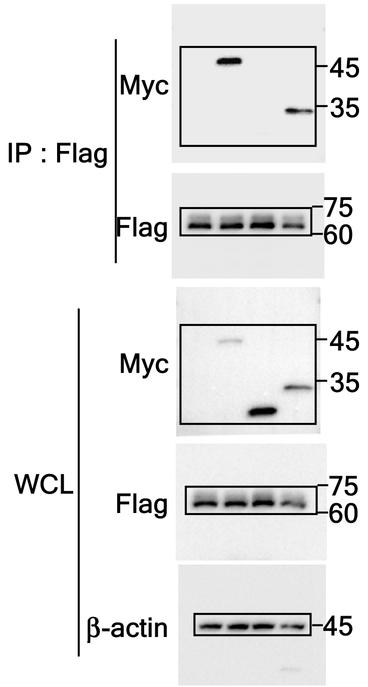

B. Fig 3C

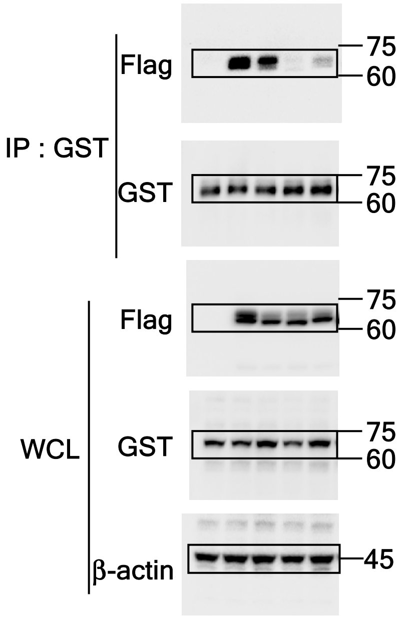

C. Fig 3D

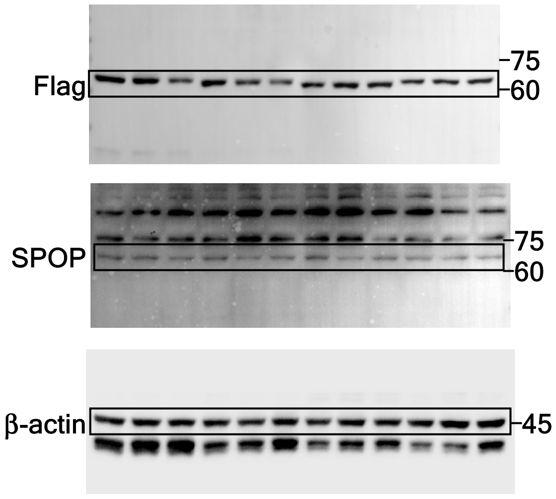

D. Fig 3E

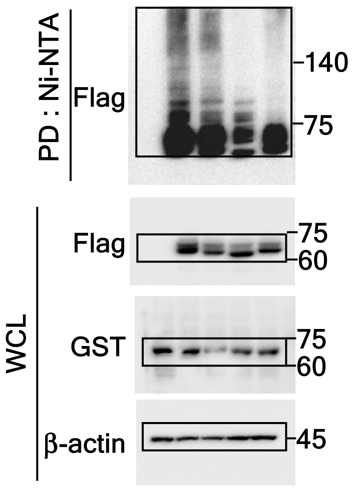

Whole blots Fig. 4 by Lee

A. Fig 4A

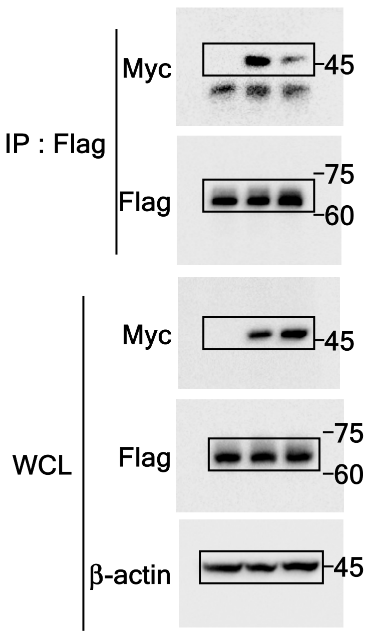

B. Fig 4C

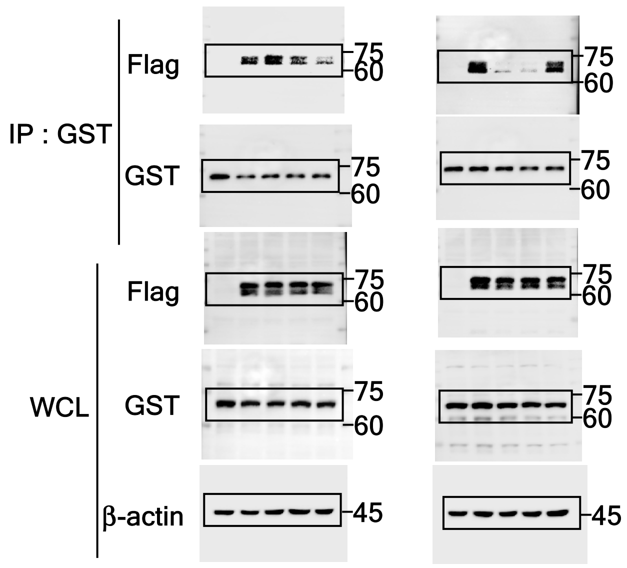

C. Fig 4D

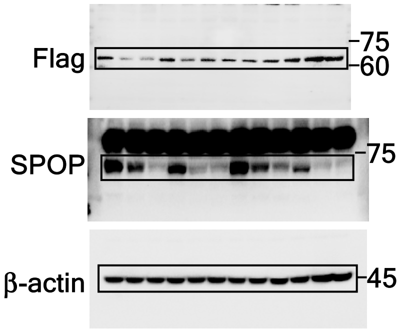

D. Fig 4E

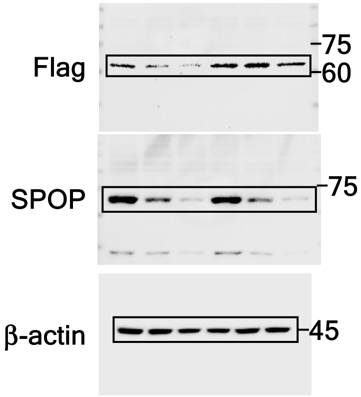

E. Fig 4F

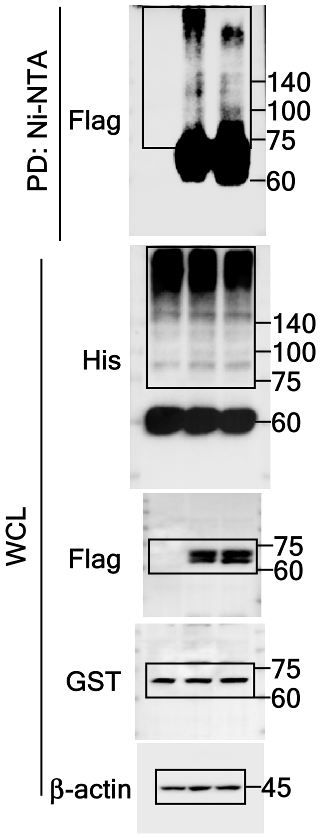

Whole blots Fig. 5 by Lee

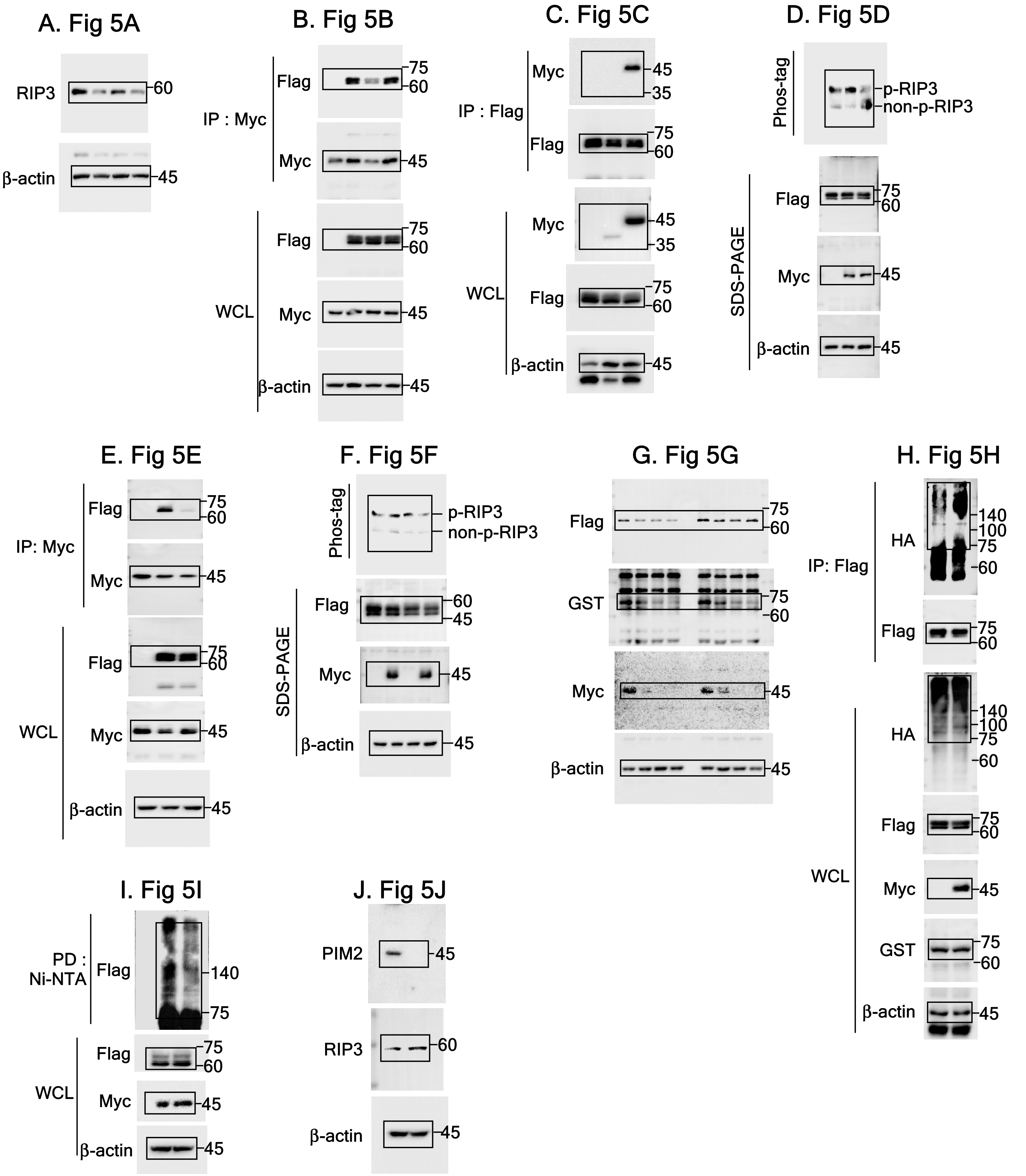

Whole blots Fig. 6 by Lee

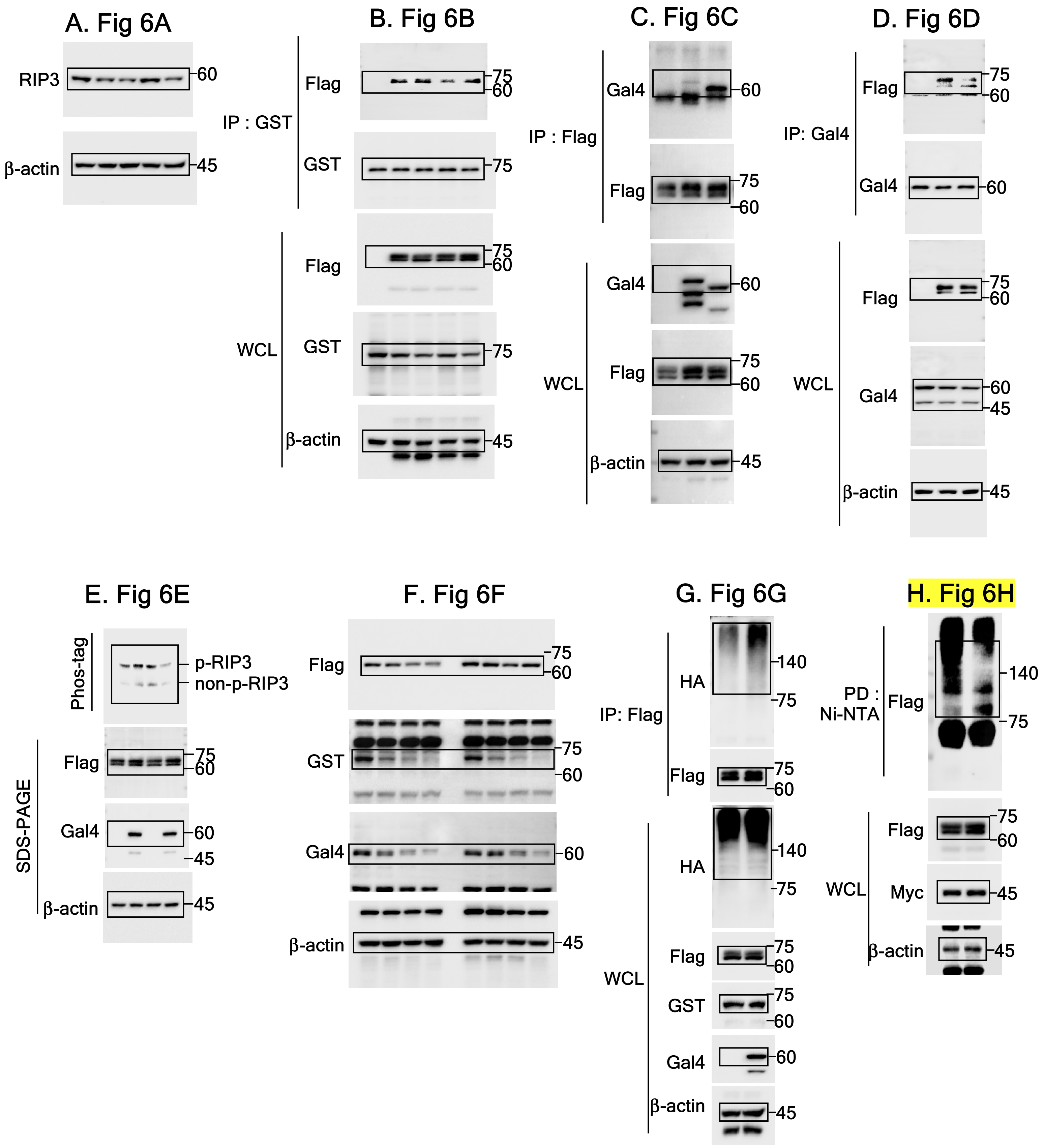

Whole blots Fig. 7 by Lee

A. Fig 7A

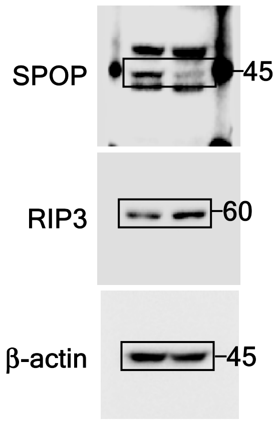

B. Fig 7D

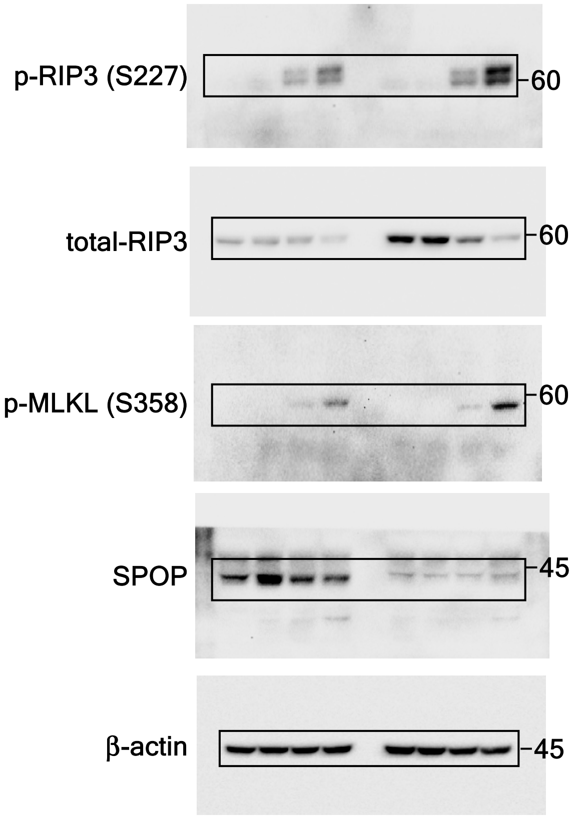

C. Fig 7E

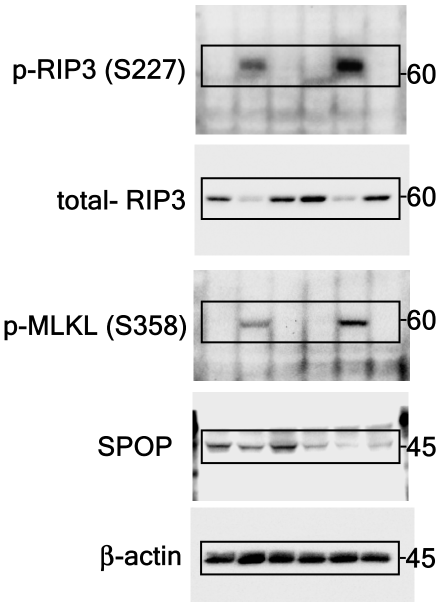

D. Fig 7F

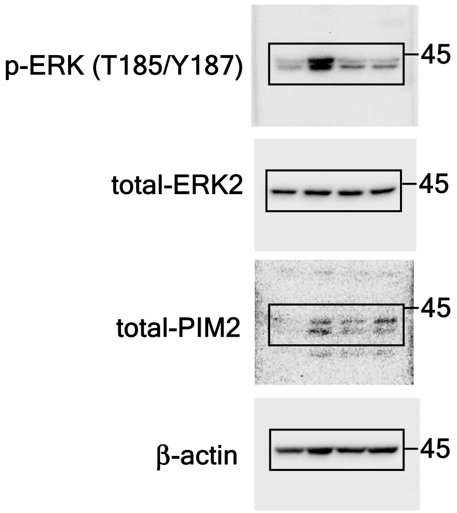

E. Fig 7G

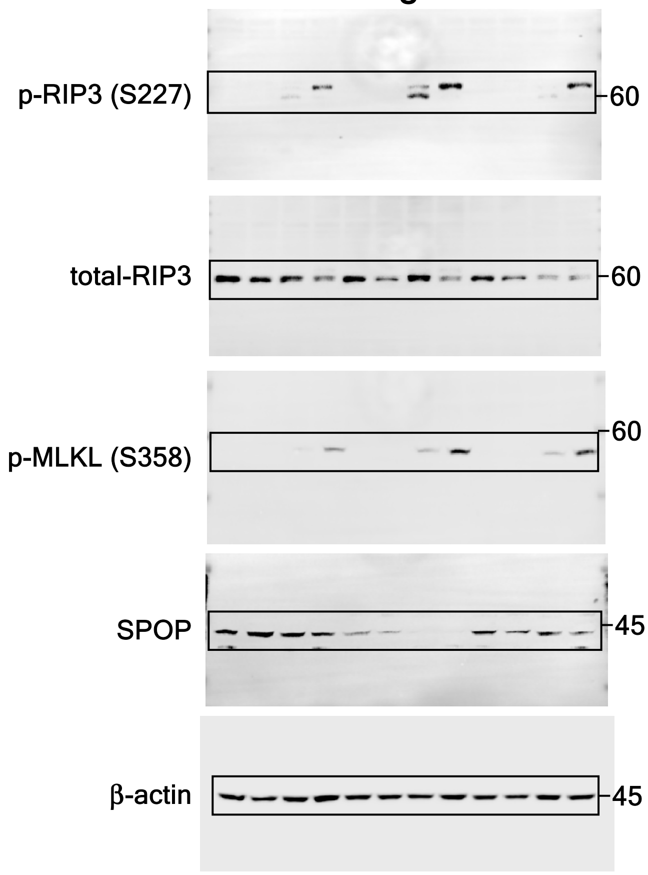

Supplementary Whole blots Fig. 1 by Lee

A. Fig S1A

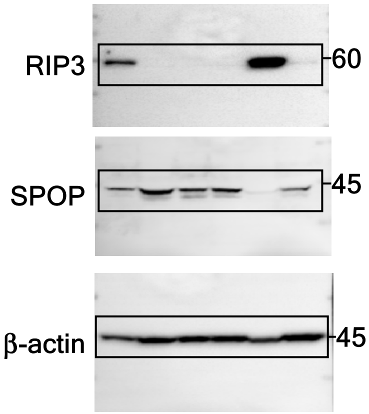

B. Fig S1B

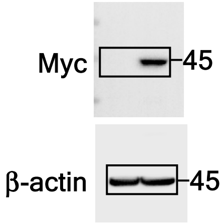

C. Fig S1C

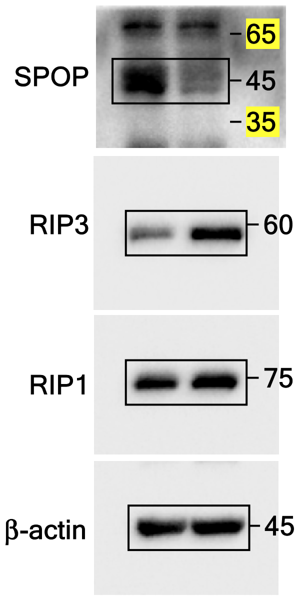

D. Fig S1D

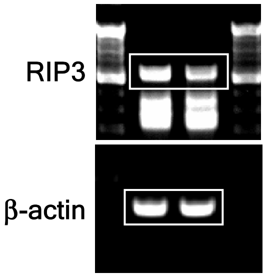

E. Fig S1E

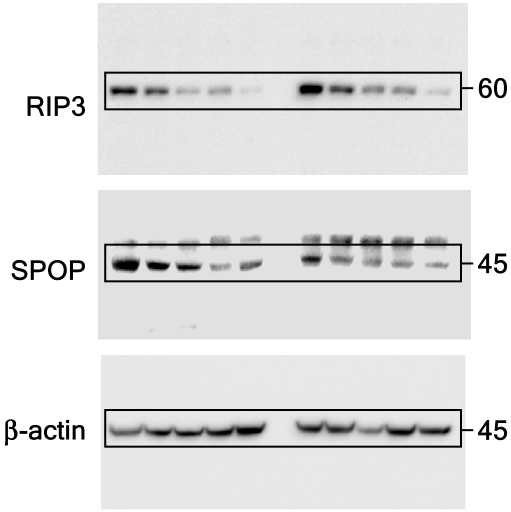

Supplement: Supplementary file 3 — Supplementary Material 3 [file 18_2024_5487_MOESM3_ESM.pdf]
