# Supplementary material for: SPOP-mediated RIPK3 destabilization desensitizes LPS/sMAC/zVAD-induced necroptotic cell death
Source: Cell Mol Life Sci. 2024 Nov 14;81(1):451. doi: 10.1007/s00018-024-05487-7 (PMC11564579; doi:10.1007/s00018-024-05487-7)
Supplement: Supplementary file 4 — Supplementary Material 4 [file 18_2024_5487_MOESM4_ESM.pdf]

English-Language Research Paper Editing Service

Issuance: NU-240927-03
Date: September 27, 2024

Prof. Yong-Yeon Cho
1BK21-4th, College of Pharmacy, The Catholic University of Korea, 43, Jibong-ro, Wonmi-gu, Bucheon-si, Gyeonggi-do 14662, Republic of Korea

Dear Prof. Cho,

It is a great pleasure to assist you in editing your manuscript.

The manuscript entitled **'SPOP-mediated RIPK3 destabilization desensitizes LPS/sMAC/zVAD-induced necroptotic cell death'** was proofread and edited by Mark B., one of the English editors at NURISCO.

NURISCO is a professional English editing company which has been working for scholars of major universities and various prestige English journals for more than 28 years. We select a specific native speaker with related major and English editing skill to improve both English and major related style of a particular manuscript.

Yours truly,

NURISCO
